# Development of potent pan‐coronavirus fusion inhibitors with a new design strategy

**DOI:** 10.1002/mco2.666

**Published:** 2024-07-28

**Authors:** Yuanmei Zhu, Zhongcai Gao, Xiaoli Feng, Lin Cheng, Nian Liu, Chao Liu, Shaowei Han, Qiaojiang Yang, Qingcui Zou, Huihui Chong, Zheng Zhang, Minghua Li, Gengshen Song, Yuxian He

**Affiliations:** ^1^ NHC Key Laboratory of Systems Biology of Pathogens National Institute of Pathogen Biology and Center for AIDS Research Chinese Academy of Medical Sciences and Peking Union Medical College Beijing China; ^2^ Research Institute of Youcare Pharmaceutical Group Co., Ltd Beijing China; ^3^ Kunming National High‐level Biosafety Research Center for Non‐Human Primates Center for Biosafety Mega‐Science Kunming Institute of Zoology Chinese Academy of Sciences Kunming Yunnan China; ^4^ Institute of Hepatology National Clinical Research Center for Infectious Disease Shenzhen Third People's Hospital The Second Affiliated Hospital, School of Medicine, Southern University of Science and Technology Shenzhen Guangdong China

**Keywords:** coronavirus, fusion inhibitor, lipopeptide, SARS‐CoV‐2

## Abstract

Development of potent and broad‐spectrum drugs against severe acute respiratory syndrome coronavirus 2 (SARS‐CoV‐2) remains one of the top priorities, especially in the cases of the emergence of mutant viruses and inability of current vaccines to prevent viral transmission. In this study, we have generated a novel membrane fusion‐inhibitory lipopeptide IPB29, which is currently under clinical trials; herein, we report its design strategy and preclinical data. First, we surprisingly found that IPB29 with a rigid linker between the peptide sequence and lipid molecule had greatly improved α‐helical structure and antiviral activity. Second, IPB29 potently inhibited a large panel of SARS‐CoV‐2 variants including the previously and currently circulating viruses, such as Omicron XBB.5.1 and EG.5.1. Third, IPB29 could also cross‐neutralize the bat‐ and pangolin‐isolated SARS‐CoV‐2‐related CoVs (RatG13, PCoV‐GD, and PCoV‐GX) and other human CoVs (SARS‐CoV, MERS‐CoV, HCoV‐NL63, and HCoV‐229E). Fourth, IPB29 administrated as an inhalation solution (IPB29‐IS) in Syrian hamsters exhibited high therapeutic and preventive efficacies against SARS‐CoV‐2 Delta or Omicron variant. Fifth, the pharmacokinetic profiles and safety pharmacology of IPB29‐IS were extensively characterized, providing data to support its evaluation in humans. In conclusion, our studies have demonstrated a novel design strategy for viral fusion inhibitors and offered an ideal drug candidate against SARS‐CoV‐2 and other coronaviruses.

## Graphical abstract

Diagram of SARS‐CoV‐2 S protein and HR2‐derived lipopeptide fusion inhibitors. The inhibitors IPB24 and IPB28 are designed with a typical flexible linker (PEG4 or GSGSG), whereas the newly designed inhibitor IPB29 uses the amino acid sequence EAAAK as a rigid linker.

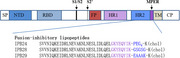

## 1 INTRODUCTION

Severe acute respiratory syndrome coronavirus 2 (SARS‐CoV‐2) infects cells through fusion of viral and cellular membranes, which is mediated by its trimeric spike (S) protein. The S1 subunit of S protein contains receptor‐binding domain (RBD) that is responsible for recognition of the human angiotensin‐converting enzyme 2 (ACE2) receptor, whereas S2 subunit mediates the membrane fusion process by assembling a six‐helical bundle (6‐HB) structure between two heptad repeat regions, HR1 and HR2.^1^, ^2^, ^3^ Structural data demonstrate that three HR1 helices form a trimeric coiled‐coil center, around which three HR2 helices are entwined in an antiparallel manner.^4^, ^5^, ^6^ It is thought that formation of 6‐HB provides the energy to drive viral and cellular membranes into close proximity for fusion and infection.

**FIGURE 1:**
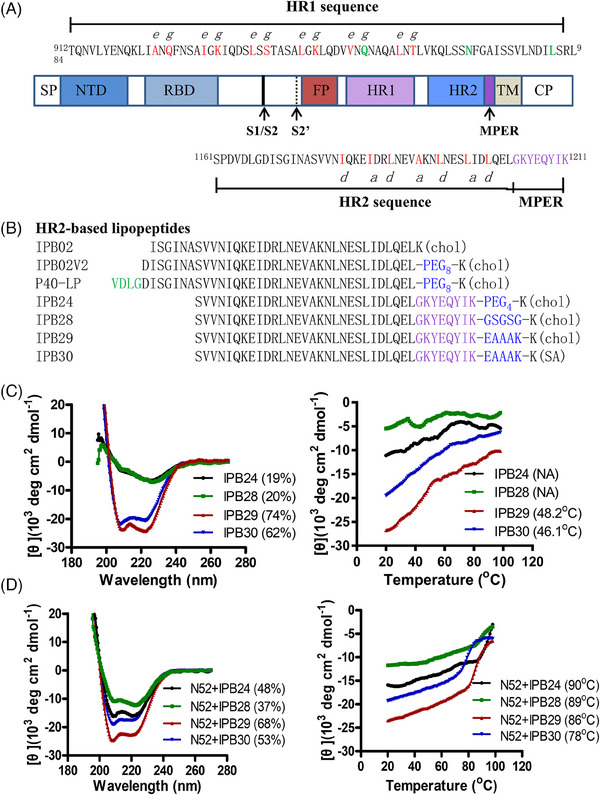
Design strategy and structural characteristics of SARS‐CoV‐2 fusion inhibitors. (A) Illustration of the S protein and HR1/HR2 sequences. SP, signal peptide; NTD, N‐terminal domain; RBD, receptor‐binding domain; FP, fusion peptide; HR1, heptad repeat 1 region; HR2, heptad repeat 2 region; MPER, membrane‐proximal external region; TM, transmembrane domain; CP, cytoplasmic peptide. The S1/S2 and S2’ cleavage sites and MPER are marked with arrow. The HR1 and HR2 sequences are listed, in which the residues that mediate the potential HR1‐HR2 interactions are colored in red. (B) HR2‐derived fusion‐inhibitory lipopeptides. Corresponding to the HR2 core sequence, the N‐terminally extended VDLG residues are marked in green and the C‐terminally extended MPER residues are colored in purple. Chol, cholesterol; PEG8 or PEG4, 8‐unit or 4‐unit polyethylene glycol; SA, stearic acid. (C and D) Structural characterization of fusion inhibitor lipopeptides by circular dichroism (CD) spectroscopy. The α‐helicity (left) and thermostability (right) of lipopeptides alone (C) or in complexes with HR1‐derived target mimic peptide N52 (D) were respectively determined with the final concentration of a lipopeptide or N52 at 10 µM.

Peptides derived from HR1 or HR2 are potent inhibitors of viral entry through blocking the assembly of 6‐HB, as exemplified by anti‐human immunodeficiency virs (HIV) drug enfuvirtide (T20), the first clinically approved viral fusion inhibitor.^7^, ^8^ This strategy has been extended to develop inhibitors against many enveloped viruses, including the emerging coronaviruses (CoVs) SARS‐CoV, MERS‐CoV, and SARS‐CoV‐2.^9^, ^10^ Since the outbreak of coronavirus disease 19 (COVID‐19), we have dedicated to characterizing the mechanism of SARS‐CoV‐2 S protein‐mediated membrane fusion and design of HR2‐based fusion‐inhibitory lipopeptides.^11^, ^12^, ^13^, ^14^, ^15^, ^16^, ^17^ As illustrated in Figure 1, IPB02 and its derivatives were designed with the HR2 core sequence, whereas P40‐LP contained an N‐terminally extended VDLG motif and IPB24 contained a membrane proximal external region (MPER). These inhibitors were characterized with very potent and broad‐spectrum activities against divergent SARS‐CoV‐2 variants, as well as other human CoVs.^12^, ^13^, ^15^, ^16^, ^17^ However, SARS‐CoV‐2 continues to evolve with larges mutations, leading to many new variants that can escape vaccines and antivirals, such as Omicron XBB.1.5 and EG.5.1; therefore, development of pan‐coronavirus inhibitors remains one of high priorities.

Previous studies have demonstrated that introducing a flexible linker or adaptor between the peptide sequence and lipid moiety is crucial for the antiviral activity of fusion‐inhibitory lipopeptides.^4^, ^12^, ^13^, ^18^, ^19^ For example, IBP02V2, P40‐LP, and IPB24 adopt an 8‐unit or 4‐unit polyethylene glycol (PEG_8_ or PEG_4_).^12^, ^13^, ^17^ In order to further characterize SARS‐CoV‐2 fusion inhibitors and develop a more efficient lipopeptide for clinical development, here we have optimized IPB24 by a rigid linker, the helix‐facilitating amino acid sequence EAAAK, leading to a new lipopeptide IPB29 with markedly improved α‐helical stability and inhibitory activity. IPB29 has been formulated as an inhalation solution (IPB29‐IS) and is currently under phase II/III clinical trials in China (ChiCTR2300068170, ChiCTR2300075467); it has been also approved for clinical investigation by the United States Food and Drug Administration (US FDA) and Australian therapeutic Goods Administration (TGA). Herein, we report the data from its design and preclinical characterizations.

## 2 RESULTS

### 2.1 Design of SARS‐CoV‐2 fusion inhibitor lipopeptides containing a rigid adaptor

All the previously reported viral lipopeptide‐based fusion inhibitors were exclusively designed with a flexible linker between the peptide sequence and lipid molecule, mostly the short PEG linker or glycine‐serine (GS) linker or their combination.^4^, ^12^, ^13^, ^17^, ^19^ For the first time, here we compared the functionalities of the flexible linker and rigid linker in designing of fusion‐inhibitory lipopeptides. Three new lipopeptides, IPB28 using a flexible “GSGSG” linker, IPB29 and IPB30 using a rigid “EAAAK” linker, were synthesized (Figure 1B), and they were characterized along with IPB24, which has an identical HR2 sequence but with a PEG_4_ linker. As determined by circular dichroism (CD) spectroscopy, IPB24 and IPB28 alone displayed an α‐helical content of ∼19–20% and their melting temperature (*T*_m_) values could not be defined, which suggested that they were largely in a random secondary structure; in a sharp contrast, IPB29 and IPB30 alone exhibited the α‐helical contents of 74 and 62%, respectively, and they had *T*_m_ values of 48.2 and 46.1°C, respectively (Figure 1C,D). When mixed with an HR1‐derived target‐mimic peptide N52, the IPB24, IPB28, IPB29, and IPB30 complexes showed the α‐helical contents of 48, 37, 68, and 53%, respectively and *T*_m_ values of 90, 89, 86, and 78°C, respectively. Therefore, the helix‐promoting EAAAK adaptor could render lipopeptide‐based fusion inhibitors with enhanced α‐helicity and thermostability.

### 2.2 IPB29 exhibits greatly improved inhibitory activity against SARS‐CoV‐2 infection

**FIGURE 2:**
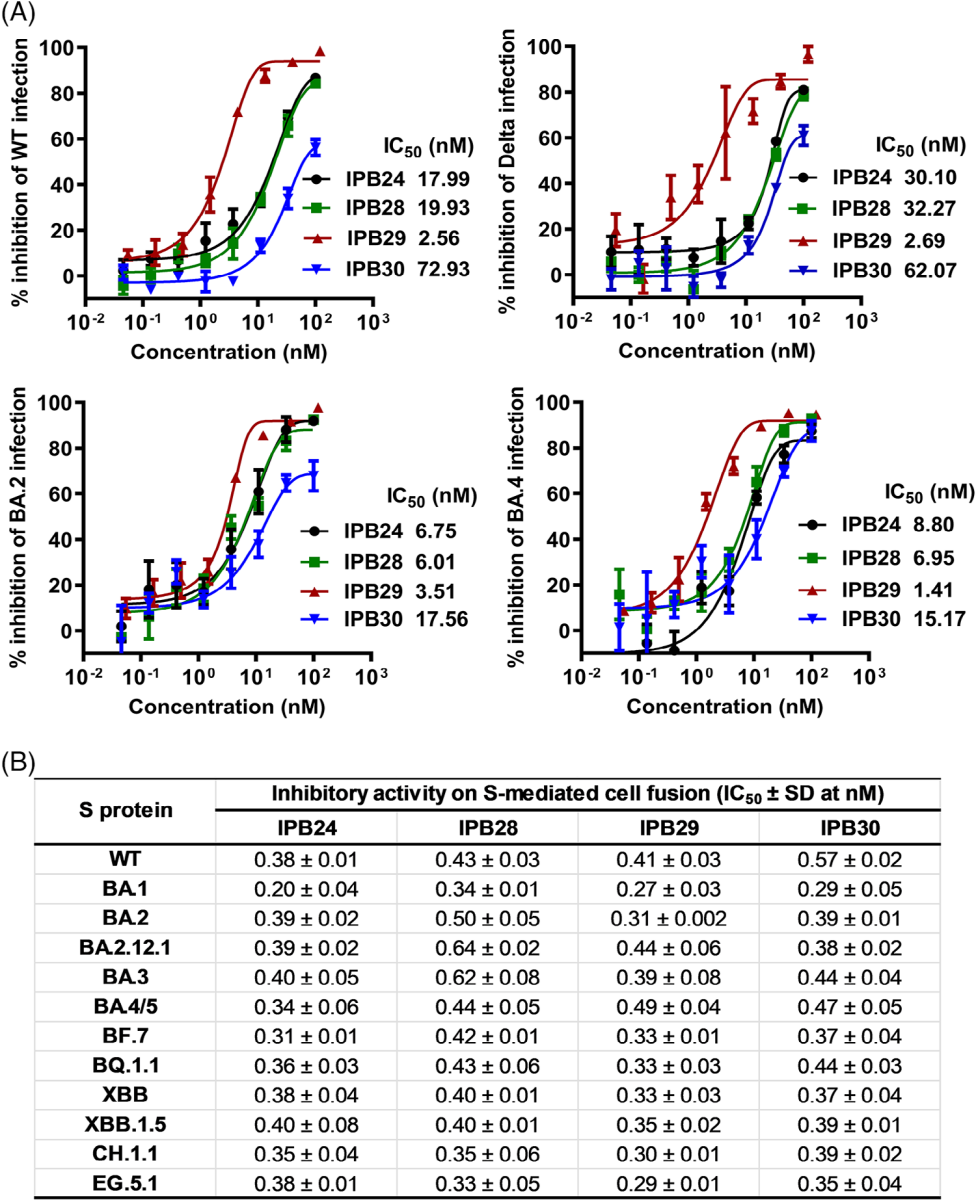
Antiviral activities of SARS‐CoV‐2 fusion inhibitor lipopeptides. The inhibitory activities of lipopeptides against live SARS‐CoV‐2 infections (A) and against S protein‐mediated cell fusion (B) were respectively determined; data are expressed as means ± standard deviations (SD).

We were intrigued to characterize the antiviral activities of the newly designed lipopeptides. To this end, several live SARS‐CoV‐2 strains, including the ancestral Wuhan‐Hu‐1 isolate (WT) and three variants of concern (Delta, Omicron BA.2, and Omicron BA.4), were applied in a focus reduction neutralization test (FRNT) in Vero E6 cells. As shown in Figure 2A, IPB24, PB28, IPB29, and IPB30 potently inhibited WT virus with the 50% inhibitory concentration (IC_50_) values of 17.99, 19.93, 2.56, and 72.93 nM, respectively; inhibited Delta virus with the IC_50_ values of 30.1, 32.27, 2.69, and 62.07 nM, respectively; inhibited Omicron BA.2 virus with the IC_50_ values of 6.75, 6.01, 3.51, and 17.56 nM, respectively; and inhibited Omicron BA.4 virus with the IC_50_ values of 8.8, 6.95, 1.41, and 15.17 nM, respectively.

We also characterized the activities of the four lipopeptides in blocking S protein‐mediated cell–cell fusion by a dual‐split protein (DSP)‐based cell fusion assay. As shown in Figure 2B, IPB24, PB28, IPB29, and IPB30 exhibited comparable inhibitory activities against the S proteins derived from the WT virus and eleven Omicron variants (BA.1, BA.2, BA2.12.1, BA.3, BA.4/5, BF.7, BQ.1.1, XBB, XBB.1.5, CH.1.1, and EG.5.1), with the IC_50_ values at subnanomolar ranges.

### 2.3 IPB29 is a highly potent inhibitor of SARS‐CoV‐2 variants and other CoVs

Given that a large number of SARS‐CoV‐2 variants emerged and caused the previous COVID‐19 pandemics worldwide, we constructed two large panels of pseudoviruses (PsV) carrying the corresponding mutant S proteins. The panel 1 viruses included the previously circulated variants (D614G, Alpha, Beta, Gamma, Delta, Lamda) and several mutants with specific substitutions (K417N, E484K, N501Y, P681R, N501Y/Δ69‐70, and N501Y/P681H); the panel 2 viruses represent divergent Omicron sublineages, including the recently emerged BF.7, BQ.1.1, XBB, XBB.1.5, and CH.1.1 variants. As determined by a single‐cycle infection assay (Table 1), IPB24, PB28, IPB29, and IPB30 inhibited the panel 1 viruses with the average IC_50_ values of 5.48, 6.39, 0.93, and 3.88 nM, respectively, in 293T/ACE2 cells and of 3.62, 4.54, 0.81, and 2.4 nM, respectively, in Huh‐7 cells. And the four lipopeptides inhibited the panel 2 viruses with average IC_50_ values of 1.75, 2.53, 0.34, and 2.46 nM, respectively, in 293T/ACE2 cells and of 1.36, 1.77, 0.28, and 1.54 nM, respectively, in Huh‐7 cells.

**TABLE 1:** Inhibitory activities of novel lipopeptides against diverse SARS‐CoV‐2 variants (IC_50_ ± SD, nM).^a^

	Pseudovirus on 293T/ACE2				Pseudovirus on Huh‐7			
Pseudovirus	IPB24	IPB28	IPB29	IPB30	IPB24	IPB28	IPB29	IPB30
Panel 1								
WT	5.51 ± 0.27	6.91 ± 0.21	0.57 ± 0.004	4.19 ± 0.05	2.43 ± 0.07	2.88 ± 0.05	0.53 ± 0.001	2.77 ± 0.12
D614G	6.17 ± 0.02	7.26 ± 0.05	0.77 ± 0.05	4.57 ± 0.16	3.15 ± 0.06	4.23 ± 0.17	0.74 ± 0.07	2.47 ± 0.10
Alpha	5.94 ± 0.10	6.12 ± 0.21	1.13 ± 0.04	3.11 ± 0.02	5.42 ± 0.30	4.29 ± 0.39	0.73 ± 0.10	2.05 ± 0.07
Beta	5.65 ± 0.01	5.75 ± 0.16	0.89 ± 0.00	2.41 ± 0.27	3.29 ± 0.76	4.21 ± 0.26	0.65 ± 0.16	1.90 ± 0.10
Gamma	6.41 ± 0.09	5.45 ± 0.02	0.53 ± 0.05	2.97 ± 0.23	3.95 ± 0.71	3.82 ± 0.28	0.57 ± 0.002	2.31 ± 0.47
Delta	4.94 ± 0.03	6.30 ± 0.47	0.79 ± 0.02	3.57 ± 0.28	3.46 ± 0.13	4.34 ± 0.23	0.56 ± 0.05	2.17 ± 0.01
Lamda	6.57 ± 0.01	7.86 ± 0.35	1.14 ± 0.02	4.81 ± 0.09	4.10 ± 0.69	5.36 ± 1.37	0.86 ± 0.10	3.45 ± 0.16
K417N	6.04 ± 0.01	6.49 ± 0.09	1.10 ± 0.06	4.07 ± 0.02	3.93 ± 0.84	5.33 ± 0.43	1.30 ± 0.25	1.78 ± 0.27
E484K	5.61 ± 0.17	8.46 ± 0.39	0.97 ± 0.03	3.91 ± 0.06	4.84 ± 0.13	5.98 ± 0.04	0.99 ± 0.16	2.40 ± 0.48
N501Y	5.24 ± 0.54	6.11 ± 0.18	1.25 ± 0.01	3.50 ± 0.26	3.47 ± 0.57	5.03 ± 0.03	0.97 ± 0.07	2.12 ± 0.03
P681R	4.31 ± 0.22	4.54 ± 0.32	0.79 ± 0.01	3.93 ± 0.15	2.09 ± 0.09	3.71 ± 0.19	0.75 ± 0.08	1.95 ± 0.17
N501Y/△69‐70	4.96 ± 0.10	6.12 ± 0.07	1.20 ± 0.13	5.56 ± 0.12	4.51 ± 0.47	5.34 ± 0.54	1.03 ± 0.01	3.37 ± 0.06
N501Y/P681H	3.84 ± 0.13	5.68 ± 0.40	1.01 ± 0.10	3.84 ± 0.19	2.48 ± 0.11	4.55 ± 0.31	0.83 ± 0.06	2.41 ± 0.63
Mean	5.48	6.39	0.93	3.88	3.62	4.54	0.81	2.4
Panel 2								
BA.1	4.51 ± 0.15	4.51 ± 0.19	0.47 ± 0.03	1.78 ± 0.09	2.56 ± 0.47	2.46 ± 0.24	0.46 ± 0.02	1.46 ± 0.09
BA.2	1.40 ± 0.32	3.02 ± 0.08	0.32 ± 0.02	2.72 ± 0.51	1.17 ± 0.16	1.48 ± 0.36	0.16 ± 0.06	1.17 ± 0.34
BA.2.12.1	1.50 ± 0.34	3.02 ± 0.33	0.31 ± 0.18	2.73 ± 0.17	1.13 ± 0.01	1.73 ± 0.35	0.26 ± 0.04	1.40 ± 0.53
BA.3	1.40 ± 0.07	2.69 ± 0.22	0.33 ± 0.03	3.29 ± 0.16	1.54 ± 0.04	2.17 ± 0.52	0.14 ± 0.02	1.28 ± 0.37
BA.4/5	1.12 ± 0.17	2.21 ± 0.43	0.23 ± 0.04	3.41 ± 0.33	1.08 ± 0.11	1.69 ± 0.22	0.2 ± 0.020	1.27 ± 0.31
BF.7	1.81 ± 0.13	1.71 ± 0.71	0.34 ± 0.04	1.65 ± 0.26	1.35 ± 0.26	1.6 ± 0.03	0.42 ± 0.14	2.09 ± 0.23
BQ.1.1	1.37 ± 0.3	2.08 ± 0.5	0.46 ± 0.14	1.98 ± 0.19	1.17 ± 0.04	1.69 ± 0.06	0.38 ± 0.14	1.86 ± 0.15
XBB	1.61 ± 0.14	1.98 ± 0.25	0.25 ± 0.03	2.66 ± 0.23	1.34 ± 0.26	1.77 ± 0.46	0.29 ± 0.02	1.4 ± 0.06
XBB.1.1.5	1.55 ± 0.19	2.13 ± 0.3	0.33 ± 0.08	1.94 ± 0.21	1.24 ± 0.11	1.65 ± 0.03	0.22 ± 0.01	1.71 ± 0.4
CH.1.1	1.2 ± 0.12	1.91 ± 0.07	0.33 ± 0.01	2.48 ± 0.54	1.04 ± 0.05	1.46 ± 0.11	0.28 ± 0.02	1.77 ± 0.08
Mean	1.75	2.53	0.34	2.46	1.36	1.77	0.28	1.54

^a^ Samples were tested in triplicate, the experiments were repeated three times, and data are expressed as means ± SD.

**FIGURE 3:**
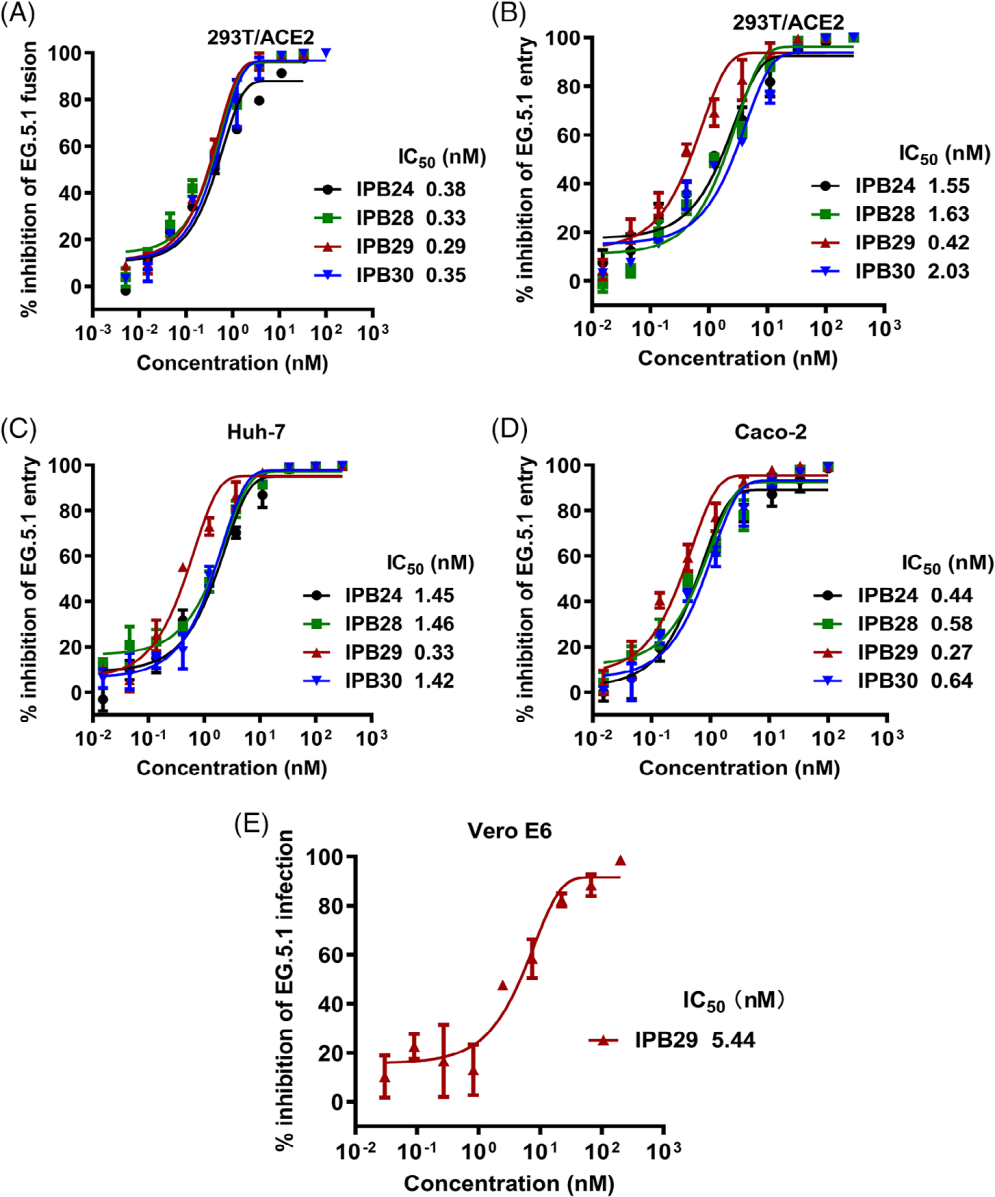
Inhibitory activity of SARS‐CoV‐2 fusion inhibitor lipopeptides against Omicron EG.5.1 variant. The inhibitory activities of lipopeptides against EG.5.1 S protein‐mediated cell fusion (A), pseudovirus infection in 293T/ACE2 (B), Huh‐7 (C), or Caco‐2 (D) cells, and live virus infection in Vero E6 cells were respective determined; data are expressed as means ± SD.

After we achieved the inhibitory data above, Omicron EG.5.1 variant emerged and has dominated the current pandemic; thus, we continued to characterize the lipopeptides. As shown, each of inhibitors maintained the potent activities in inhibiting EG.5.1 cell fusion (Figure 3A), PsV entry in 293T/ACE2, Huh‐7, or Caco‐2 cells (Figure 3C,D), and live virus infection in Vero E6 cells (Figure 3E). Taken together, these results demonstrated that IPB29 was the most potent inhibitor of divergent SARS‐CoV‐2 variants, and that Omicron sublineages even exhibited increased susceptibility to the fusion inhibitors.

Our previous studies showed that SARS‐CoV‐2 HR2‐derived fusion inhibitors could effectively inhibit other human CoVs, including the highly pathogenic SARS‐CoV and MERS‐CoV, as well as HCoV‐NL63 and HCoV‐229E, two viruses that cause mild respiratory infection.^12^, ^13^, ^17^ Herein, we first examined the inhibitory activities of IPB24, PB28, IPB29, and IPB30 on three SARS‐CoV‐2‐related CoVs, namely the bat‐isolated RaTG13 and pangolin‐isolated PCoV‐GD and PCoV‐GX. As determined by PsV‐based single‐cycle infection assay, IPB29 displayed the highest potency in inhibiting three SARS‐CoV‐2‐related CoVs in 293T/ACE2 cells among the lipopeptides tested (Table 2). Similarly, IPB29 inhibited the infection of SARS‐CoV PsV in Huh‐7 cells more efficiently. As anticipated, all four lipopeptides also neutralized the PsV of MERS‐CoV, HCoV‐NL63, and HCoV‐229E efficiently.

**TABLE 2:** Inhibitory activities of novel lipopeptides against SARS‐CoV‐2‐related CoVs and other human CoVs.^a^

		IC_50 _± SD (nM)			
Pseudovirus	Target cells	IPB24	IPB28	IPB29	IPB30
Bat RaTG13	293T/ACE2	5.08 ± 1.37	4.94 ± 1.97	1.23 ± 0.56	2.33 ± 0.79
PCoV‐GD	293T/ACE2	7.92 ± 0.38	7.10 ± 0.03	1.35 ± 0.11	7.30 ± 0.62
PCoV‐GX	293T/ACE2	5.83 ± 0.28	5.15 ± 1.04	1.25 ± 0.26	6.76 ± 1.28
SARS‐CoV	Huh‐7	18.81 ± 1.27	18.69 ± 3.98	6.17 ± 0.57	65.29 ± 0.44
MERS‐CoV	Huh‐7	70.36 ± 7.96	140.36 ± 49.23	100.32 ± 17.70	719.73 ± 30.5
HCoV‐NL63	Huh‐7	186.97 ± 49.45	304.93 ± 38.94	80.78 ± 8.81	712.77 ± 17.40
HCoV‐229E	Huh‐7	287.97 ± 54.82	748.35 ± 19.63	309.3 ± 12.63	2054.17 ± 109.13

^a^ Samples were tested in triplicate, the experiments were repeated three times, and data are expressed as means ± SD.

### 2.4 IPB29 displays low cell toxicity and high in vitro stability

To validate the in vitro antiviral potencies of the inhibitors determined above, we measured their cytotoxicity in the target cells used in the inhibition assays. As shown in Figure S1A, each of the lipopeptides exhibited the median cytotoxic concentration (CC_50_) values at two digit macromolar (µM) levels in 293T/ACE2, Huh‐7 or Vero E6 cells. Specifically, IPB29 exhibited the CC_50_ values of 23.94 µM in 293T/ACE2, 22.75 µM in Huh‐7, and 45.27 µM in Vero E6 cells. These results indicated that the lipopeptides possess relatively low cytotoxicity and high therapeutic selection index (CC_50_/IC_50_).

Herein, we also determined the in vitro stability of IPB29 along with IPB24, including their sensitivities to the treatment of proteolytic enzymes, human serum, human liver microsome, or high temperature. After two lipopeptides were treated with different experimental conditions, their inhibitory activities against D614G PsV were examined by the single‐cycle infection assay. As shown in Figure S1B, both IPB24 and IPB29 were highly resistant to the digestions of proteinase K, trypsin, and chymotrypsin; however, IPB24 showed markedly decreased antiviral activities after incubation with the human serum and liver microsome or storage at 37°C overtime, whereas the inhibitory activities of IPB29 were almost unchanged. Especially, while IPB24 was highly sensitive to the incubation of 20% human serum at 37°C, IPB29 could largely tolerate the treatment at the same condition. Thus, IPB29 has excellent in vitro stability and druggability.

### 2.5 Nebulized IPB29 exhibits high therapeutic efficacies against Delta variant

**FIGURE 4:**
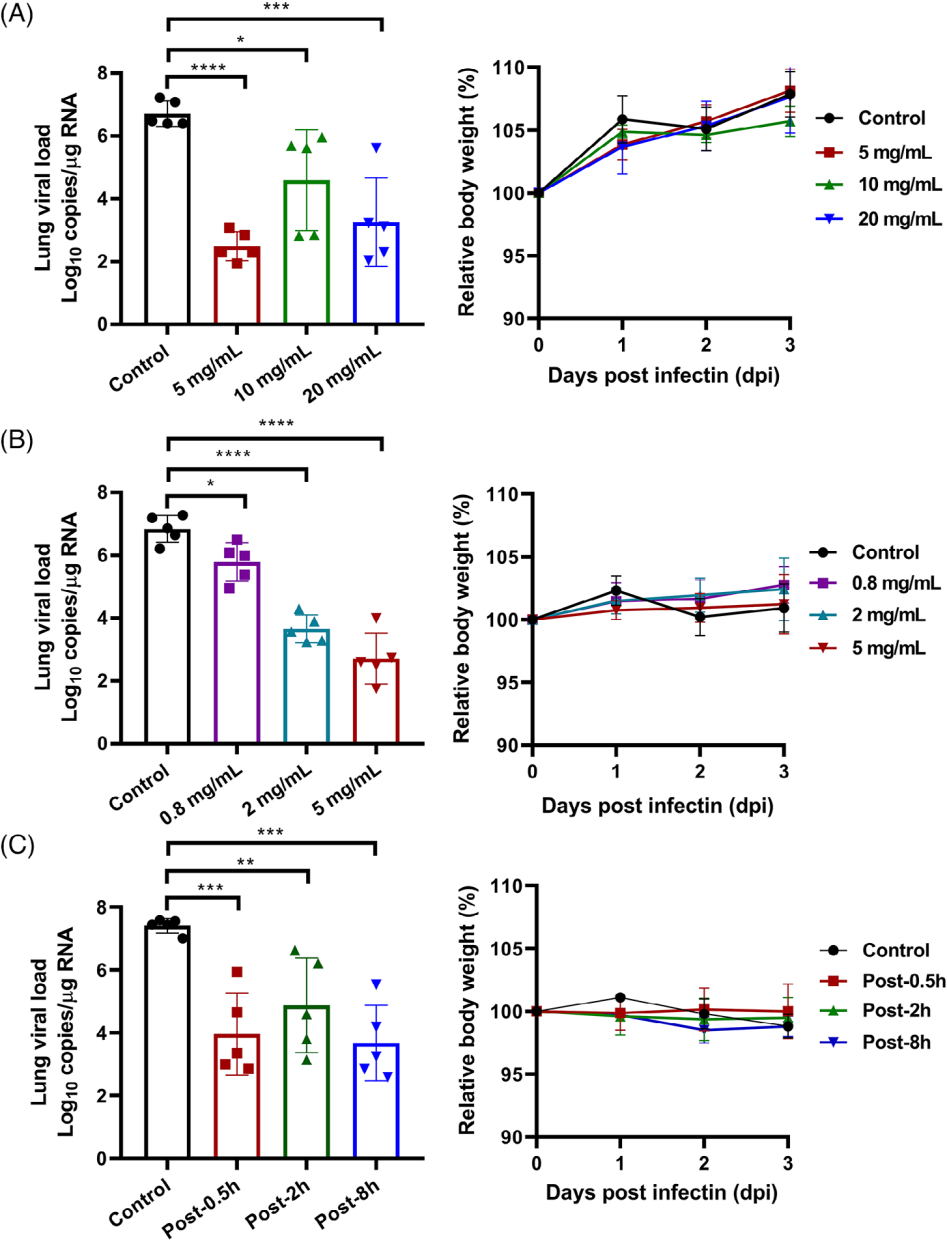
Therapeutic efficacy of IPB29 inhalation solution against SARS‐CoV‐2 Delta variant. (A) The first dose‐exploratory study, in which 20 Syrian hamsters were challenged by SARS‐CoV‐2 Delta variant and after 0.5 h the hamsters were treated with 5, 10, 20 mg/mL of IPB29‐IS or vehicle for 30 min through nebulization, once daily for 3 days. (B) The second dose‐exploratory study, in which 20 hamsters were challenged by the Delta variant and after 0.5 h the hamsters were treated with IPB29‐IS at 0.8, 2, 5 mg/mL or vehicle for 30 min through nebulization, once daily for 3 days. (C) The time‐exploratory study, in which 30 hamsters infected by the Delta virus were nebulized for 30 min with 5 mg/mL of IPB29‐IS at 0.5, 2, or 8 h postinfection or with the vehicle at 0.5 h postinfection, once daily for 3 days. The lung viral loads (left) and body weights (right) of hamsters were monitored for comparisons.

Considering the characteristics of SARS‐CoV‐2 infection and lung disease, IPB29 was formulated as an inhalation solution (IPB29‐IS). Initially, a Syrian hamster infection model of SARS‐CoV‐2 Delta variant with an intranasal challenge of 1 × 10^4^ TCID_50_/animal was established and two dose exploratory studies were conducted to determine the effective dose of the drug. In the first study, 20 male hamsters were randomly divided into four groups of five animals each: vehicle control group (water for injection), high‐dose group (20 mg/mL), middle‐dose group (10 mg/mL), and low‐dose group (5 mg/mL). After 0.5 h of viral challenge, nebulized inhalation of different doses of IPB29‐IS was performed for 30 min and was administrated once daily for 3 consecutive days. The hamsters were then dissected and lung tissues were taken for viral load testing. As shown in Figure 4A, the lung viral loads of all dosing groups were significantly lower than that of vehicle control group; and surprisingly, the 5 mg/mL treated group exhibited the largest reduction with the Log10 vRNA copies/g from 6.71 to 2.49. Thus, the second dose exploration study was carried out to examine the efficacy of reduced IPB29‐IS dosage in 20 male hamsters (five/group). After being challenged with the virus, the hamsters were nebulized with different doses of IPB29‐LP (5 mg/mL group, 2 mg/mL group, or 0.8 mg/mL group) or vehicle for 30 min, once daily for 3 days. Comparing with the vehicle control group, three groups of drug‐treated animals had significantly reduced viral loads in the lung tissues, and the 5 mg/mL group showed the highest efficacy with its Log10 vRNA copies decreased from 6.84 to 2.71 (Figure 4B). As monitored, the body weights of hamsters of all test article groups increased, but no significant difference was observed between the dosing groups and the control group.

We also evaluated the therapeutic effect of IPB29‐IS administered at different time points after SARS‐CoV‐2 infection. For this, 30 male hamsters were divided into six groups of five animals each and were nebulized for 30 min with the vehicle at 0.5 h postinfection (control group) or with 5 mg/mL of IPB29‐IS at 0.5 h (post‐0.5 h group), 2 h (post‐2 h group), or 8 h (post‐8 h group) postinfection, once daily for 3 days. As shown in Figure 4C, the lung viral loads of three IPB29‐IS groups were significantly lower than that of vehicle control, and no significant difference for body weight changes was observed.

### 2.6 Nebulized IPB29 has high therapeutic and preventive efficacies against Omicron variant

**FIGURE 5:**
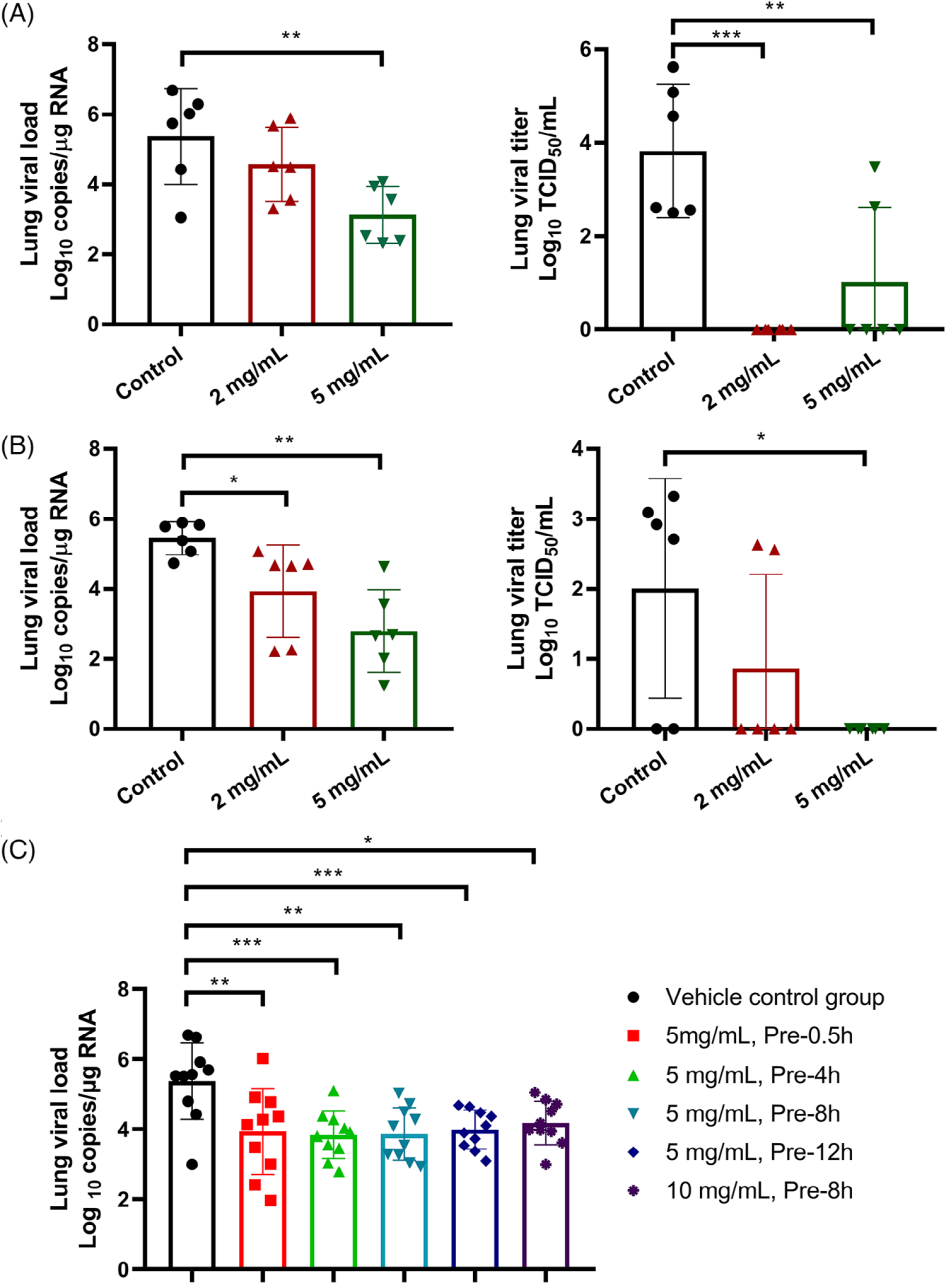
Therapeutic and preventive efficacies of IPB29 inhalation solution against SARS‐CoV‐2 Omicron variant. (A and B) The therapeutic efficacy of IPB29‐IS against Omicron BA.2 variant. Thirty‐six Syrian hamsters were challenged by the virus at 0.5 h prior to the treatment; the hamsters were then nebulized with 2 or 5 mg/mL of IPB29‐IS or vehicle control for 30 min, once daily for 3 days (A) or 5 days (B). The lung viral loads were determined for both RNA copies (left panels) and TCID_50_ (right panels). (C) The preventive efficacy of IPB29‐IS against Omicron BA.2 variant. Sixty Syrian hamsters were nebulized for 30 min with a single dose of 5 mg/mL IPB29‐IS at 0.5, 4, 8, or 12 h or 10 mg/mL IPB29‐IS at 8 h or vehicle control at 0.5 h prior to the virus challenge. The lung viral loads were measured on day 2 after challenge.

After we finished the animal challenge studies above, Omicron variants emerged and have now become dominant viruses. Herein, we evaluated the therapeutic efficacy of IBB29‐IS with a hamster infection model of Omicron BA.2 variant at an intranasal challenge dose of 1 × 10^4^ TCID_50_/hamster. Thirty‐six hamsters were randomly divided into three groups of 12 animals each (half of each sex): vehicle control group, 2 mg/mL, or 5 mg/mL dosing group of IBB29‐IS. At 0.5 h postinfection, the animals were nebulized for 30 min, once daily for 3 or 5 days. Comparing with that of the control animals, the lung viral loads of the 5 mg/mL group, but not the 2 mg/mL group, significantly reduced after treatment 3 days (Figure 5A, left); however, the lung viral loads of both IBB29‐IS groups significantly reduced after treatment 5 days (Figure 5B, left). When the infectious viruses in the lung tissues were determined at TCID_50_, it revealed that the lung viral titers after treatment 3 days were undetectable in all animals of the 2 mg/mL group and in four animals of the 5 mg/mL group (Figure 5A, right); after treatment 5 days, the lung viral titers could not be detected in all animals of the 5 mg/mL group and in four animals of the 2 mg/mL group (Figure 5B, right). Similarly, no significant difference for body weights was observed between each test article group and the vehicle control group (Figure S2A,B).

Next, we conducted a study to evaluate the preventive efficacy of IPB29‐IS against Omicron BA.2 infection. Sixty hamsters were randomly divided into six groups of 10 animals each (half of each sex), nebulized for 30 min with a single dose of 5 mg/mL IPB29‐IS at 0.5 h (pre‐0.5 h group), 4 h (pre‐4 h group), 8 h (pre‐8 h group), and 12 h (pre‐12 h group) or water for injection (vehicle control) at 0.5 h before the virus challenge of 1 × 10^4^ TCID_50_/hamster. An additional 10 mg/mL IPB29‐IS group was also carried out 8 h prior to the challenge. Lung tissues were sampled for viral load testing on day 2 after challenge, and the results showed that IPB29‐IS‐pretreated hamsters had significantly reduced viral loads in lung tissues compared with the vehicle control group (Figure 5C). Different from the treatment studies, the body weights of IPB29‐IS‐pretreated hamsters significantly increased relative to those of the control group, but no difference for body temperature was observed (Figure S2C,D). Taken together, our studies with 166 Syrian hamsters demonstrated that nebulized IPB29‐IS solution possessed high in vivo therapeutic and prophylactic efficacies.

### 2.7 Pharmacokinetics and drug metabolism of IPB29 inhalation solution

Nonclinical pharmacokinetics (PK) of IPB29‐IS was first evaluated in 48 Golden hamsters (Table S1). After a single oronasal inhalation of IPB29‐IS (Table S2), with average delivery doses at 0.854 or 2.432 mg/kg (in a single induction cartridge inhalation of IPB29‐IS of 2 and 5 mg/mL for 30 min), the *C*_max_ and AUC_last_ of IPB29 in the hamster plasma increased with increasing dose, and no significant gender difference was found for the AUC_last_ in the plasma and lung (Tables S3 and S4). The concentration ratio of lung tissue to plasma was 446.86 in males and 357.72 in females (Table S5), and the AUC_last_ ratio of lung tissue to plasma was 550.82 in males and 409.40 in females (Table S6).

A total of 24 Beagle dogs were then used for the PK analysis. They were randomly assigned to four groups (three/sex/group), including the intravenous group (1 mg/kg), inhalation groups of low dose (average delivery dose of 1.566 mg/kg), middle dose (average delivery dose of 4.885 mg/kg), and high dose (average delivery dose of 13.303 mg/kg) with a single administration for all groups (Table S7). In the dose range of 1.566 to 13.303 mg/kg, the *T*_max_ of IPB29 in dogs ranged from 6 to 10 h, the *C*_max_ and AUC_last_ of IPB29 in dog plasma increased with increasing dose (Tables S8–S11). After a single inhalation with average doses at 1.566, 4.885, 13.303 mg/kg, in relative to the single intravenous administration at 1 mg/kg, the calculated absolute bioavailability of IPB29 ranged from 0.15 to 0.20% in males and 0.11 to 0.23% in females (Table S12).

The PK and tissue distribution studies of IPB29‐IS were conducted in SD rats (Table S13). In the inhalation dose range of 3.896 to 32.401 mg/kg, the *T*_max_ of IPB29 ranged from 3 to 4.5 h, the plasma exposure (AUC_last_) of IPB29 increased with increasing dose (Tables S14 and S15). After a single inhalation with delivery doses at 3.896, 11.259, 32.401 mg/kg, in relative to the single intravenous administration at 3 mg/kg, the calculated absolute bioavailability based on AUC_last_ of IPB29 ranged from 0.02 to 0.03%. There was no significant gender difference in AUC_last_ in plasma, lung or trachea of animals, and the AUC_last_ ratios of lung to plasma were 3308.35 in males and 3614.25 in females, the AUC_last_ ratios of trachea to plasma were 1123.22 in males and 1128.26 in females (Table S16).

At concentrations of 0.1, 1, and 10 µM, the plasma protein binding of IPB29 incubated for 6 h in the plasma of ICR/CD‐1 mouse, SD rat, Beagle dog, Cynomolgus monkey, and human was about 99.9% cross species. No significant concentration dependence and species difference were observed (Table S17). After IPB29 at 1 µM was incubated in plasma of ICR/CD‐1 mouse, SD rat, Beagle dog, Cynomolgus monkey, and human, respectively, for 6 h, the remaining percentages of the parent drug were 104, 109, 109, 107, and 109%, respectively, indicating that IPB29 was very stable in plasma of five species. After IPB29 was incubated with hepatocytes from ICR/CD‐1 mouse, SD rat, Beagle dog, Cynomolgus monkey, and human at 37°C for 120 min, the remaining percentages of the parent drug was 61.5, 64.4, 7.04, 50.7, and 43.1%, respectively (Table S18).

In the hepatocytes of ICR/CD‐1 mouse, SD rat, Beagle dog, Cynomolgus monkey, and human, IPB29 had a metabolic half‐life of 165, 191, 29.7, 175, and 106 min, respectively, and its intrinsic clearance rate was 0.00839, 0.00727, 0.0467, 0.00792, and 0.0131 mL/min/10^6^ cells, respectively. The metabolite profiles of IPB29 were investigated, indicating that they were all mediated by hydrolysis (Table S18).

### 2.8 General toxicity and genotoxicity of IPB29 inhalation solution

To support the IND application, general toxicology studies, a genotoxicity study panel and a systemic anaphylaxis test (Table S19) were conducted in compliance with the US FDA Good Laboratory Practice (GLP) regulations. In the 7‐day dose range finding study in SD rats, 5, 10, and 20 mg/mL of IPB29‐IS (actual delivered doses were 4.07 ± 0.44, 12.04 ± 1.06, and 72.46 ± 6.56 mg/kg) administered daily to rats by repeated inhalation caused no significant local or systemic toxicity; its no‐observed‐adverse‐effect level (NOAEL) was 72.46 ± 6.56 mg/kg, the highest dose tested. The mean systemic exposure (AUC_last_) in each dose group increased with the increase of dose on day 1 and day 7. There was no apparent accumulation when animals were repeatedly dosed for 7 days compared with the first dose (Table S20).

In the 4‐week toxicity study in SD rats, administration of 5, 10, and 20 mg/mL of IPB29‐IS (actual delivered doses of 5.264, 19.246, or 49.570 mg/kg) showed no respiratory irritation, systemic toxicity, or target organ toxicity; the NOAEL was 49.570 ± 5.205 mg/kg. At this dose, *C*_max_ and AUC_last_ were 39.60 ng/mL and 273.11 h ng/mL in males and 38.25 ng/mL and 343.77 h ng/mL in females on day 28 (Table S21).

In the 7‐day dose range finding study in Beagle dogs, 5, 10, and 20 mg/mL IPB29‐IS (actual delivered doses of 2.154, 5.800, and 13.269 mg/kg) was administered to dogs by repeated inhalation once daily for 7 days. At doses up to 13.269 ± 2.860 mg/kg, there was no obvious local or systemic toxicity observed, giving the NOAEL of 13.269 ± 2.860 mg/kg. The mean systemic AUC_last_ of IPB29 in animals on day 1 and day 7 increased along with increasing dose. After 7‐days repeated inhalation administration of IPB29‐IS at low, mid and high doses in dogs, the mean *C*_max_ ratio (day 7/day 1) of IPB29 ranged from 1.01 to 2.87, and the mean AUC_last_ ratio (day 7/day 1) ranged from 1.30 to 2.96, indicating no apparent accumulation (Table S22).

In the 4‐week toxicity study in dogs, administration of 5, 10, and 20 mg/mL of IPB29‐IS (actual delivered doses of 2.475, 5.568, 14.798 mg/kg) showed no systemic toxicity, respiratory irritation, or target organs of toxicity; its NOAEL was 14.798 mg/kg. The mean AUC_last_ of IPB29 increased along with increasing of the dose on day 1 and day 28, and no statistically significant difference in plasma between female and male animals. After 28‐day repeated dose administration, the mean AUC_last_ ratios (day 28/day 1) of IPB29 ranged from 0.44 to 3.47 (Table S23).

A battery of genotoxicity studies, including a bacterial reverse mutation assay (Ames), an in vitro mammalian chromosomal aberration test, and an in vivo micronucleus assay, were conducted, showing that IPB29‐IS was negative and did not cause active systemic anaphylaxis in guinea pigs.

We further verified that IPB29 had no significant effects on the biological functions of 45 targets selected (except hERG and AR) in the secondary pharmacodynamics studies, and its risk of off‐target was low on hERG and AR. IPB29 inhibited the hERG currents of HEK293 cells with an average IC_50_ value greater than 12.79 µmol/L (Table S24). There were no test article‐related effects on the central nervous system following a single inhalation at target dosage of 5.657, 21.401, or 59.260 mg/kg IPB29‐IS in SD rats, and no indications of adverse effects on the respiratory systems or the cardiovascular safety in Beagle dogs following inhalation at target dosage of 2.423, 6.777, and 12.094 mg/kg.

## 3 DISCUSIION

The global COVID‐19 pandemic has lasted more than 3 years and caused nearly seven million confirmed deaths (https://covid19.who.int/). The pathogen SARS‐CoV‐2 continues to spread with evolutionary mutations, leading to many variants that challenge the efficacies of current vaccines and neutralizing antibodies.^20^, ^21^, ^22^, ^23^, ^24^ The Omicron variant emerged as the fifth variant of concern after Alpha, Beta, Gamma, and Delta variants, having the largest mutations, higher transmissibility and more prone to immune evasion.^25^, ^26^ Currently, Omicron has already evolved into distinct sublineages, including BA.1, BA.2, BA.3, BA.4, BA.5, BF.7, BQ.1.1, XBB, XBB.1.5, CH.1.1, EG.5.1, and so on. To curtail the pandemic, the development of potent and broad‐spectrum anti‐SARS‐CoV‐2 drugs remains one of the top priorities, especially in the cases of current vaccines cannot effectively prevent viral transmission and the failure of antibody‐based therapies.

Membrane fusion is an essential step for enveloped viruses and inhibitors that can impede the functionality of viral fusion protein have been extensively explored.^3^, ^27^, ^28^ Peptides derived from the fusion protein HR2 domain have strong antiviral activity through blocking 6‐HB structure.^29^, ^30^ Comparing with unmodified template peptides, lipid‐modified lipopeptides possess greatly improved pharmaceutical properties, including binding affinity, inhibitory activity, and in vivo stability.^17^, ^31^, ^32^, ^33^ While a native HR2 peptide can block the cell surface fusion pathway, instead of endosomal pathway, lipopeptides also enables activity against viruses that do not fuse until they have been taken up through endocytosis. Also, a lipopeptide can bind to both viral and cell membranes, which greatly elevates the local concentration of inhibitors thereby enhancing the antiviral activity dramatically.^32^, ^34^, ^35^ In a design strategy, a flexible linker or adaptor between the peptide sequence and lipid molecule is generally required for lipopeptides; and in practice, a short PEG linker or GS sequence or their combination has been widely applied.^12^, ^13^ As exemplified by the reported SARS‐CoV‐2 fusion inhibitors, IPB02‐ and IPB24‐based lipopeptides used a PEG_8_ or PEG_4_ linker^12^, ^13^; EK1‐based lipopeptides (EK1L1C, EK1C4, and EK1‐C16) used GSG or a tandem of GSGSG and PEG_4_ ^4^, ^36^, ^37^; SARS_HRC_–PEG_4_–chol and its dimer format used the GSGSGC segment, with the cysteine side chain as a nucleophilic handle to append a cholesterol unit via an intervening PEG_4_ linker.^18^, ^19^ In this study, we dedicated to develop more efficient SARS‐CoV‐2 fusion inhibitors on the basis of our previous achievements. For the first time, we applied a rigid linker as a designing strategy of lipopeptides. As shown, the PEG_4_ in IPB24 was replaced by the helix‐facilitating amino acid sequence EAAAK, which created the lipopeptides IPB29 and IPB30 with dramatically increased α‐helicity and thermostability relative to the flexible linker‐embedded lipopeptides (IPB24 and IPB28). As anticipated, IPB29 was a more potent inhibitor than IPB24 and IPB28 in inhibiting diverse replication‐competent SARS‐CoV‐2 isolates and S protein‐pseudotypes; and it also potently inhibited SARS‐CoV‐2‐related CoVs isolated from bat (RaTG13) or pangolin (PCoV‐GD and PCoV‐GX) and other human CoVs (SARS‐CoV, MERS‐CoV, HCoV‐NL63, and HCoV‐229E). By referring to the previously reported lipopeptides IPB21 and IPB22,^13^ it is conceivable that IPB30 described in this study is more active than the inhibitors that are modified by the same fatty acid molecule. IPB29 displayed relatively lower cytotoxicity and higher resistance to the treatment of human serum, liver microsome, and high temperature, providing additional evidence to support its druggability. Based on the CD spectroscopy data, here we speculate that the secondary structure of the lipopeptide inhibitors might correlated with their chemical stability. Taken together, we conclude that the EAAAK linker can render lipopeptide‐based fusion inhibitors with improved pharmaceutical profiles, and this strategy in designing fusion inhibitors against other enveloped viruses, such as HIV, influenza virus, and respiratory syncytial virus, should be exploited.

Since the SARS‐CoV‐2 outbreak, tremendous efforts have been devoted to the development of drugs that target different stages of viral life‐cycle, generating a group of inhibitors approved for urgent use, and of which neutralizing antibody therapy is a mainstay.^38^, ^39^ For example, bamlanivimab/etesevimab, casirivimab/imdevimab (REGN‐COV2), tixagevimab/cilgavimab (Evusheld), and BRII‐196/BRII‐198 are cocktails of two noncompeting, neutralizing human antibodies that target the spike RBD, whereas sotrovimab (VIR‐7831) and bebtelovimab (LY‐CoV1404) recognize the relatively conserved RBD epitopes thus being developed as a single antibody therapy.^40^, ^41^ Unfortunately, the continuously emerging SARS‐CoV‐2 variants have dramatically reduced the effectiveness of the antibody‐based therapies, leading to the withdrawn of their emergency use authorization.^41^, ^42^, ^43^ In contrast, the HR2‐based fusion inhibitors could fully maintain the antiviral efficacy against various SARS‐CoV‐2 variants, even being highly active inhibitors of pan‐sarbecoviruses, as demonstrated by the data described previously and in the current study.^15^, ^16^, ^44^, ^45^, ^46^ Different from the action mechanism of other classes of antivirals such as paxlovid and molnupiravir, which target the key enzymes (3CL or RdRP) required for viral replication and exert their inhibition after the virus enters the targeting cells, the membrane fusion inhibitors block the early step of infection^38^, ^39^, ^40^, ^41^; thus, we think that IPB29 has multiple advantages in the treatment and prevention of SARS‐CoV‐2 infection.

So far, no fusion inhibitor‐based drugs have been approved for use to treat or prevent COVID‐19 in clinical settings. In this racetrack, we formulated IPB29 as nebulized solution (IPB29‐IS) and systemically investigated its PK, drug metabolism, safety pharmacology, and antiviral efficacy in animal models. IPB29‐IS exhibited high therapeutic and prophylactic efficacies against SARS‐CoV‐2 infection in Syrian hamsters, which provided solid data to support its clinical development. The results have also validated the nebulization treatment strategy for human coronaviruses and other respiratory infections. In an advanced stage, IPB29 is currently under phase II/III clinical trials. As reported, EK1 peptide and P315V3 lipopeptide have also been advanced to clinical trials.^46^, ^47^ Therefore, we are looking forward to the coming of a new class of drugs that can be used in the battle against the SARS‐CoV‐2 pandemic. Definitely, we will continue our efforts to develop novel viral fusion inhibitors that can overcome the limitations of the current lipopeptides, including their in vivo half‐life, route of administration, as well as the cost effectiveness. In this regard, we have produced two highly potent, long‐lasting HIV fusion inhibitor drugs being evaluated under clinical trials,^31^ which would also inform the advantages and disadvantages of the technical platforms.

## 4 MATERIALS AND METHODS

### 4.1 Plasmids and cells

Plasmids encoding the SARS‐CoV‐2 wild‐type (WT) and mutant S proteins, as well as the S proteins of SARS‐CoV, MERS‐CoV, HCoV‐NL63 and HCoV‐229E, and 293T/ACE2 cells were previously constructed in our laboratory.^13^ The plasmids encoding the S proteins of SARS‐CoV‐2 variants were kindly provided by Linqi Zhang at the Tsinghua University (Beijing, China). The plasmids encoding the S proteins of the bat‐isolated RaTG13 and the pangolin‐isolated PCoV‐GD and PCoV‐GX were a gift from Zhaohui Qian at the Institute of Pathogen Biology, Chinese Academy of Medical Sciences (Beijing, China). HEK293T, Huh‐7, Caco‐2, and Vero E6 cells were purchased from the American Type Culture Collection (Rockville, MD, USA).

### 4.2 Production of peptides and lipopeptides

Peptides were synthesized by a standard solid‐phase 9‐flurorenylmethoxycarbonyl method as described previously.^13^ Three cholesterol‐conjugated lipopeptides (IPB24, IPB28, and IPB29) were prepared by amidation of a C‐terminal lysine side chain with cholesteryl succinate monoester, in which 4‐unit PEG (PEG_4_), GSGSG or EAAAK was introduced as a flexible or rigid linker. IPB30 was prepared with the template peptide containing a C‐terminal lysine with a 1‐(4,4‐dimethyl‐2,6‐dioxocyclohexylidene)ethyl side chain‐protecting group, enabling the conjugation of fatty acid C18. All peptides were purified by reverse‐phase high‐performance liquid chromatography to homogeneity of greater than 95% and their molecular weights were characterized by mass spectrometry.

### 4.3 CD spectroscopy

The α‐helicity and thermostability of lipopeptide inhibitors as well as their complexes with an HR1‐derived target‐mimic peptide N52 were measured by CD spectroscopy as described previously.^13^ In which, lipopeptides were diluted with a final concentration of 10 µM in phosphate‐buffered saline (pH 7.2). CD spectrum was obtained on Jasco spectropolarimeter model J‐815, and melting temperature (*T*_m_) was defined as the midpoint of thermal unfolding transition.

### 4.4 Inhibition of authentic SARS‐CoV‐2 infection

To measure the inhibitory activity of fusion inhibitors on replicative SARS‐CoV‐2 infection, a FRNT was conducted in a certified biosafety level‐3 facility with minor modification.^48^ In brief, serially threefold dilutions of lipopeptide were added to Vero E6 cells seeded in 96‐well plates and incubated for 1 h at 37°C. Then, 200 foci forming unit of a live virus were added to the culture wells and incubated for 1 h at 37°C. After replacing the supernatants with medium containing 1.6% carboxymethylcellulose and 2% FBS, the plates were incubated at 37°C for an additional 24 h. The cells were then fixed with 4% paraformaldehyde solution, permeabilized with Perm/Wash buffer (BD Biosciences, Franklin Lakes, NJ, USA) containing 0.1% Triton X‐100, followed by incubation with HRP‐conjugated anti‐SARS‐CoV‐2 N protein antibody P301‐F7. The reactions were developed using KPL TrueBlue peroxidase substrate (Seracare Life Sciences Inc., Cambridge, MA, USA). The number of virus foci was quantified using an EliSpot reader (Cellular Technology Ltd., Cleveland, Ohio, USA). The percent inhibition of virus infection and 50% inhibitory concentration (IC_50_) of inhibitors were calculated using GraphPad Prism software (GraphPad Software Inc., San Diego, CA, USA).

### 4.5 Cell–cell fusion assay

The inhibitory activities of lipopeptides on S protein‐mediated cell fusion were measured by a DSP‐based cell fusion assay as described previously,^17^ in which the effector 293T cells were cotransfected with S‐expressing plasmid and DSP_1–7_ plasmid, whereas the target 293T/ACE2 cells were transfected with DSP_8–11_ plasmid. The lipopeptides were serially diluted at threefold.

### 4.6 Single‐cycle infection assay

The inhibitory activities of lipopeptides against various SARS‐CoV‐2 PsV in 293T/ACE2, Caco‐2, or Huh‐7 cells were determined by a single‐cycle infection assay as described previously.^17^ PsV was packaged in HEK293T cells by cotransfecting the S‐expressing plasmid and a lentiviral backbone plasmid (pNL4‐3.luc.RE). The 50% tissue culture infectious dose (TCID_50_) of PsV was determined in target cells by the Reed‐Muench method. The lipopeptides were serially diluted at threefold, and 500 TCID_50_ of PsV were applied.

### 4.7 Formulation of IPB29 inhalation solution

IPB29 was formulated as an inhalation solution (IPB29‐IS) containing IPB29 peptide, citric acid, sodium citrate, mannitol, and water for injection, and it was supplied as sterile lyophilized powder in a single‐dose transparent glass vial fitted with a rubber stopper and a combined aluminum‐plastic cap. Each vial contained 20 mg of IPB29 based on the drug substance molecular formula (C_251_H_414_N_60_O_74_), reconstituted with 2 mL of water for injection prior to use. Stability studies on three batches of drug product demonstrated that IPB29‐IS remained chemically and physically stable during one month of storage at accelerated condition (25 ± 2°C/60% ± 5% RH) and one month at long term stability condition (5 ± 3°C).

### 4.8 Nebulization of IPB29 inhalation solution

The nebulizer system (PARI TurboBOY, Germany) and a customized transparent induction box with a three‐cage stainless isolation cage was connected through a flexible hose and used for the inhalation administration of IPB29‐IS in Syrian hamsters. The compressor of the nebulizer system had a mean output of 7.2 L/min during nebulization and the aerosol had a mean mass median aerodynamic diameter of 3.5 µm, and the percentage of particles smaller than 5 µm was 67%. To start the nebulization, 5 mL of IPB29‐IS or solvent control was firstly added in an atomizing cup, three hamsters were placed in the isolation cage in the induction box, then turning on the nebulizer system power and continuing into dry running of the cup for about 30 min, so that the hamsters could inhale the drug aerosol through normal breathing. The hamsters were removed and put back into the original cage for further feeding.

### 4.9 Quantitation of viral loads in lung tissues

Total RNA was extracted from homogenized hamster tissues using a TRIzol reagent (Thermo Fisher Scientific). SARS‐CoV‐2 RNAs were detected by one‐step RT‐PCR using a THUNDERBIRD Probe One‐Step qRT‐PCR (TOYOBO, Japan) following the manufacturer's protocols. The primers targeting the N protein were used, including 5′‐GGGGAACTTCTCCTGCTAGAAT‐3′, 5′‐CAGACATTTTGCTCTCAAGCTG‐3′, probe 5′‐FAM‐TTGCTGCTGCTTGACAGATT‐TRMRA‐3′. Serial dilutions of the SARS‐CoV‐2 RNA reference standard (National Institute of Metrology, China) were used in each run, in parallel to calculate copy numbers in each sample on ViiA 7 Real‐Time PCR System (Applied Biosystems).

### 4.10 PK of IPB29 inhalation solution

Nonclinical PK of IPB29‐IS was evaluated in Golden hamsters by a single inhalation administration and in Beagle dogs by a single inhalation or intravenous administration. A PK and tissue distribution study of IPB29‐IS was also conducted in SD rats by intravenous and inhalation administrations. The method details were described in the Supporting Information. An LC–MS/MS method was developed and validated for the quantification of IPB29 concentrations in rat and dog plasma. The linearity range in rat plasma was 0.5–50 ng/mL and the lower limit of quantification (LLOQ) was 0.5 ng/mL. The linearity range in dog plasma was 1–100 ng/mL and the LLOQ was 1 ng/mL. The fully validated LC–MS/MS method was well used for the PK and toxicokinetic (TK) studies of IPB29 in SD rats and Beagle dogs. An LC–MS/MS method was developed and validated for the quantification of IPB29 concentrations in rat lung tissues. The developed methods were validated with a linear range of 1–100 ng/mL. The fully validated LC–MS/MS method was well used for the determination of IPB29 in rat lung tissues. The precision, accuracy and other parameters results met the acceptance criteria. In addition, respective ELISA method was validated for determination of anti‐IPB29 antibody in SD rat serum and Beagle dog serum, and the stability of anti‐IPB29 antibody in SD rat serum and Beagle dog serum in different conditions was assessed.

### 4.11 Toxicity of IPB29 inhalation solution

To support the IND application, general toxicology studies, a genotoxicity study panel and a systemic anaphylaxis test were systemically conducted in compliance with the US FDA GLP regulations. The method details were described in the Supporting Information. Specifically, these studies included 4‐week toxicity and TK studies in SD rats and Beagle dogs with 4‐week recovery periods; a bacterial reverse mutation assay (Ames), an in vitro chromosomal aberration test, an in vivo mammalian erythrocyte micronucleus assay in mice; and an active systemic anaphylaxis test in guinea pigs. In addition, exploratory (non‐GLP) 7‐day repeat dose range finding studies of IPB29‐IS were conducted in rats and dogs. IPB29‐IS was prepared equivalent to that proposed for the clinic. In vivo studies were conducted via inhalation, the intended route (and using the method) of administration in humans. The high dose for the general toxicity studies was based on a maximum feasible dose, using the test article at the maximum solubility (20 mg/mL) delivered over a period of time that was acceptable for repeat dose inhalation studies in the chosen species. Inhalation of clean air served as the negative control in the studies.

### 4.12 Statistical analysis

The data were analyzed using GraphPad Prism 7 software (GraphPad Software Inc.). One‐way ANOVA with Dunnett's multiple comparisons test was used to analyze the differences between the experimental groups (ns, not significant; *, *p* < 0.05; **, *p* < 0.01; ***, *p* < 0.001; ****, *p* < 0.0001), in which *p *< 0.05 is considered as a significant difference.

## AUTHOR CONTRIBUTIONS

Y. Z., Z. G., X. F., L. C., N. L., C. L., S. H., Q. Y., Q. Z., and H. C. performed the experiments. Z. Z., M. L., G. S., and Y. H. supervised the study and analyzed the data. Y. H. designed the study and wrote the paper with Y. Z. All authors have read and approved the final manuscript.

## CONFLICT OF INTEREST STATEMENT

The authors declare that there is no conflict of interest.

## Supporting information

Supporting Information

## Data Availability

The data that support the findings of this study are available from the corresponding author upon reasonable request.

## References

[1] Walls AC , Park YJ , Tortorici MA , et al. Structure, function, and antigenicity of the SARS‐CoV‐2 spike glycoprotein. Cell. 2020;181(2):281‐292. 32155444 10.1016/j.cell.2020.02.058 PMC7102599

[2] Wrapp D , Wang N , Corbett KS , et al. Cryo‐EM structure of the 2019‐nCoV spike in the prefusion conformation. Science. 2020;367(6483):1260‐1263. 32075877 10.1126/science.abb2507 PMC7164637

[3] V'Kovski P , Kratzel A , Steiner S , Stalder H , Thiel V . Coronavirus biology and replication: implications for SARS‐CoV‐2. Nat Rev Microbiol. 2021;19(3):155‐170. 33116300 10.1038/s41579-020-00468-6 PMC7592455

[4] Xia S , Liu M , Wang C , et al. Inhibition of SARS‐CoV‐2 (previously 2019‐nCoV) infection by a highly potent pan‐coronavirus fusion inhibitor targeting its spike protein that harbors a high capacity to mediate membrane fusion. Cell Res. 2020;30(4):343‐355. 32231345 10.1038/s41422-020-0305-x PMC7104723

[5] Sun H , Li Y , Liu P , et al. Structural basis of HCoV‐19 fusion core and an effective inhibition peptide against virus entry. Emerg Microbes Infect. 2020;9(1):1238‐1241. 32482145 10.1080/22221751.2020.1770631 PMC7448930

[6] Yang K , Wang C , White KI , et al. Structural conservation among variants of the SARS‐CoV‐2 spike postfusion bundle. Proc Natl Acad Sci USA. 2022;119(16):e2119467119. 35363556 10.1073/pnas.2119467119 PMC9169775

[7] Wild CT , Shugars DC , Greenwell TK , McDanal CB , Matthews TJ . Peptides corresponding to a predictive alpha‐helical domain of human immunodeficiency virus type 1 gp41 are potent inhibitors of virus infection. Proc Natl Acad Sci USA. 1994;91(21):9770‐9774. 7937889 10.1073/pnas.91.21.9770 PMC44898

[8] Lalezari JP , Henry K , O'Hearn M , et al. Enfuvirtide, an HIV‐1 fusion inhibitor, for drug‐resistant HIV infection in North and South America. N Engl J Med. 2003;348(22):2175‐2185. 12637625 10.1056/NEJMoa035026

[9] Schutz D , Ruiz‐Blanco YB , Munch J , et al. Peptide and peptide‐based inhibitors of SARS‐CoV‐2 entry. Adv Drug Deliv Rev. 2020;167:47‐65. 33189768 10.1016/j.addr.2020.11.007 PMC7665879

[10] Tang T , Bidon M , Jaimes JA , Whittaker GR , Daniel S . Coronavirus membrane fusion mechanism offers a potential target for antiviral development. Antiviral Res. 2020;178:104792. 32272173 10.1016/j.antiviral.2020.104792 PMC7194977

[11] Zhu Y , Yu D , Yan H , Chong H , He Y . Design of potent membrane fusion inhibitors against SARS‐CoV‐2, an emerging coronavirus with high fusogenic activity. J Virol. 2020;94(14):e00635‐20. 32376627 10.1128/JVI.00635-20 PMC7343218

[12] Zhu Y , Yu D , Hu Y , et al. SARS‐CoV‐2‐derived fusion inhibitor lipopeptides exhibit highly potent and broad‐spectrum activity against divergent human coronaviruses. Signal Transduct Target Ther. 2021;6(1):294. 34344868 10.1038/s41392-021-00698-x PMC8330190

[13] Yu D , Zhu Y , Jiao T , et al. Structure‐based design and characterization of novel fusion‐inhibitory lipopeptides against SARS‐CoV‐2 and emerging variants. Emerg Microbes Infect. 2021;10(1):1227‐1240. 34057039 10.1080/22221751.2021.1937329 PMC8216258

[14] Yu D , Zhu Y , Yan H , et al. Pan‐coronavirus fusion inhibitors possess potent inhibitory activity against HIV‐1, HIV‐2, and simian immunodeficiency virus. Emerg Microbes Infect. 2021;10(1):810‐821. 33847245 10.1080/22221751.2021.1917309 PMC8812798

[15] Zhu Y , Hu Y , Liu N , Chong H , He Y . Potent inhibition of diverse Omicron sublineages by SARS‐CoV‐2 fusion‐inhibitory lipopeptides. Antiviral Res. 2022;208:105445. 36265805 10.1016/j.antiviral.2022.105445 PMC9574594

[16] Zhu Y , Dong X , Liu N , et al. SARS‐CoV‐2 fusion‐inhibitory lipopeptides maintain high potency against divergent variants of concern including Omicron. Emerg Microbes Infect. 2022;11(1):1819‐1827. 35786417 10.1080/22221751.2022.2098060 PMC9310806

[17] Hu Y , Zhu Y , Yu Y , et al. Design and characterization of novel SARS‐CoV‐2 fusion inhibitors with N‐terminally extended HR2 peptides. Antiviral Res. 2023;212:105571. 36868315 10.1016/j.antiviral.2023.105571 PMC9977133

[18] Outlaw VK , Bovier FT , Mears MC , et al. Inhibition of coronavirus entry in vitro and ex vivo by a lipid‐conjugated peptide derived from the SARS‐CoV‐2 spike glycoprotein HRC domain. mBio. 2020;11(5):e01935‐20. 33082259 10.1128/mBio.01935-20 PMC7587434

[19] de Vries RD , Schmitz KS , Bovier FT , et al. Intranasal fusion inhibitory lipopeptide prevents direct‐contact SARS‐CoV‐2 transmission in ferrets. Science. 2021;371(6536):1379‐1382. 33597220 10.1126/science.abf4896 PMC8011693

[20] Cao Y , Yisimayi A , Jian F , et al. BA.2.12.1, BA.4 and BA.5 escape antibodies elicited by Omicron infection. Nature. 2022;608(7923):593‐602. 35714668 10.1038/s41586-022-04980-y PMC9385493

[21] Bowen JE , Addetia A , Dang HV , et al. Omicron spike function and neutralizing activity elicited by a comprehensive panel of vaccines. Science. 2022;377(6608):890‐894. 35857529 10.1126/science.abq0203 PMC9348749

[22] Zhang J , Xiao T , Cai Y , et al. Membrane fusion and immune evasion by the spike protein of SARS‐CoV‐2 Delta variant. Science. 2021;374(6573):1353‐1360. 34698504 10.1126/science.abl9463 PMC10763652

[23] Wang P , Nair MS , Liu L , et al. Antibody resistance of SARS‐CoV‐2 variants B.1.351 and B.1.1.7. Nature. 2021;593(7857):130‐135. 33684923 10.1038/s41586-021-03398-2

[24] Tao K , Tzou PL , Nouhin J , et al. The biological and clinical significance of emerging SARS‐CoV‐2 variants. Nat Rev Genet. 2021;22(12):757‐773. 34535792 10.1038/s41576-021-00408-x PMC8447121

[25] Fan Y , Li X , Zhang L , et al. SARS‐CoV‐2 Omicron variant: recent progress and future perspectives. Signal Transduct Target Ther. 2022;7(1):141. 35484110 10.1038/s41392-022-00997-x PMC9047469

[26] Sun C , Xie C , Bu GL , Zhong LY , Zeng MS . Molecular characteristics, immune evasion, and impact of SARS‐CoV‐2 variants. Signal Transduct Target Ther. 2022;7(1):202. 35764603 10.1038/s41392-022-01039-2 PMC9240077

[27] Yang H , Rao Z . Structural biology of SARS‐CoV‐2 and implications for therapeutic development. Nat Rev Microbiol. 2021;19(11):685‐700. 34535791 10.1038/s41579-021-00630-8 PMC8447893

[28] Plavec Z , Pohner I , Poso A , Butcher SJ . Virus structure and structure‐based antivirals. Curr Opin Virol. 2021;51:16‐24. 34564030 10.1016/j.coviro.2021.09.005 PMC8460353

[29] He Y . Synthesized peptide inhibitors of HIV‐1 gp41‐dependent membrane fusion. Curr Pharm Des. 2013;19(10):1800‐1809. 23092277 10.2174/1381612811319100004

[30] Duzgunes N , Fernandez‐Fuentes N , Konopka K . Inhibition of viral membrane fusion by peptides and approaches to peptide design. Pathogens. 2021;10(12):1599. 10.3390/pathogens10121599 PMC8709411 34959554

[31] Xue J , Chong H , Zhu Y , et al. Efficient treatment and pre‐exposure prophylaxis in rhesus macaques by an HIV fusion‐inhibitory lipopeptide. Cell. 2022;185(1):131‐144.. 34919814 10.1016/j.cell.2021.11.032

[32] Zhu Y , Chong H , Yu D , et al. Design and characterization of cholesterylated peptide HIV‐1/2 fusion inhibitors with extremely potent and long‐lasting antiviral activity. J Virol. 2019;93(11):e02312‐e02318. 30867304 10.1128/JVI.02312-18 PMC6532087

[33] Chong H , Xue J , Zhu Y , et al. Monotherapy with a low‐dose lipopeptide HIV fusion inhibitor maintains long‐term viral suppression in rhesus macaques. PLoS Pathog. 2019;15(2):e1007552. 30716118 10.1371/journal.ppat.1007552 PMC6375636

[34] Zhu Y , Li M , Liu N , et al. Development of highly effective LCB1‐based lipopeptides targeting the spike receptor‐binding motif of SARS‐CoV‐2. Antiviral Res. 2023;211:105541. 36682464 10.1016/j.antiviral.2023.105541 PMC9851916

[35] Chong H , Zhu Y , Yu D , He Y . Structural and functional characterization of membrane fusion inhibitors with extremely potent activity against HIV‐1, HIV‐2, and simian immunodeficiency virus. J Virol. 2018;92(20):e01088‐18. 30089693 10.1128/JVI.01088-18 PMC6158425

[36] Lan Q , Chan JF , Xu W , et al. A palmitic acid‐conjugated, peptide‐based pan‐CoV fusion inhibitor potently inhibits infection of SARS‐CoV‐2 omicron and other variants of concern. Viruses. 2022;14(3):549. 35336956 10.3390/v14030549 PMC8955410

[37] Zhou J , Xu W , Liu Z , et al. A highly potent and stable pan‐coronavirus fusion inhibitor as a candidate prophylactic and therapeutic for COVID‐19 and other coronavirus diseases. Acta Pharm Sin B. 2022;12(4):1652‐1661. 34367893 10.1016/j.apsb.2021.07.026 PMC8327648

[38] Talukder P , Saha A , Roy S , et al. Drugs for COVID‐19 treatment: a new challenge. Appl Biochem Biotechnol. 2023;196(6):3653‐3670. 10.1007/s12010-023-04439-4 PMC10037400 36961509

[39] Li G , Hilgenfeld R , Whitley R , De Clercq E . Therapeutic strategies for COVID‐19: progress and lessons learned. Nat Rev Drug Discov. 2023;22(6):449‐475. 37076602 10.1038/s41573-023-00672-y PMC10113999

[40] Liu M , Gan H , Liang Z , et al. Review of therapeutic mechanisms and applications based on SARS‐CoV‐2 neutralizing antibodies. Front Microbiol. 2023;14:1122868. 37007494 10.3389/fmicb.2023.1122868 PMC10060843

[41] de Almeida Oliveira A , Praia Borges Freire D , Rodrigues de Andrade A , et al. The landscape of neutralizing monoclonal antibodies (nAbs) for treatment and prevention of COVID‐19. J Pharm Innov. 2023:1‐19. 10.1007/s12247-023-09713-w PMC9943047 36843665

[42] Touret F , Giraud E , Bourret J , et al. Enhanced neutralization escape to therapeutic monoclonal antibodies by SARS‐CoV‐2 omicron sub‐lineages. iScience. 2023;26(4):106413. 36968074 10.1016/j.isci.2023.106413 PMC10015083

[43] Group AC‐TfIwC‐S . Efficacy and safety of two neutralising monoclonal antibody therapies, sotrovimab and BRII‐196 plus BRII‐198, for adults hospitalised with COVID‐19 (TICO): a randomised controlled trial. Lancet Infect Dis. 2022;22(5):622‐635. 34953520 10.1016/S1473-3099(21)00751-9 PMC8700279

[44] Xia S , Wang L , Jiao F , et al. SARS‐CoV‐2 Omicron subvariants exhibit distinct fusogenicity, but similar sensitivity, to pan‐CoV fusion inhibitors. Emerg Microbes Infect. 2023;12(1):2178241. 36748716 10.1080/22221751.2023.2178241 PMC9970205

[45] Wang X , Sun L , Liu Z , et al. An engineered recombinant protein containing three structural domains in SARS‐CoV‐2 S2 protein has potential to act as a pan‐human coronavirus entry inhibitor or vaccine antigen. Emerg Microbes Infect. 2023;12(2):2244084. 37534910 10.1080/22221751.2023.2244084 PMC10424610

[46] Wang L , Jiao F , Jiang H , et al. Fusogenicity of SARS‐CoV‐2 BA.2.86 subvariant and its sensitivity to the prokaryotic recombinant EK1 peptide. Cell Discov. 2024;10(1):6. 38191587 10.1038/s41421-023-00631-2 PMC10774434

[47] Wu L , Zheng A , Tang Y , et al. A pan‐coronavirus peptide inhibitor prevents SARS‐CoV‐2 infection in mice by intranasal delivery. Sci China Life Sci. 2023;66(10):2201‐2213. 37574525 10.1007/s11427-023-2410-5

[48] Zhou B , Cheng L , Song S , et al. Identification and application of a pair of noncompeting monoclonal antibodies broadly binding to the nucleocapsid proteins of SARS‐CoV‐2 variants including Omicron. Virol J. 2022;19(1):96. 35643510 10.1186/s12985-022-01827-w PMC9142731

