# Supplementary material for: Development of potent pan‐coronavirus fusion inhibitors with a new design strategy
Source: MedComm (2020). 2024 Jul 28;5(8):e666. doi: 10.1002/mco2.666 (PMC11283584; doi:10.1002/mco2.666)
Supplement: Supplementary file 1 — Supporting Information [file MCO2-5-e666-s001.docx]

**SUPPLEMENTARY INFORMATION**

**Development of potent pan-coronavirus fusion inhibitors with a new design strategy**

Running title: Potent pan-coronavirus fusion inhibitors

Yuanmei Zhu^1#^, Zhongcai Gao^2#^, Xiaoli Feng^3#^, Lin Cheng^4#^, Nian Liu^1^, Chao Liu^2^, Shaowei Han^2^, Qiaojiang Yang^3^, Qingcui Zou^3^, Huihui Chong^1^, Zheng Zhang^2^*, Minghua Li^3^*, Gengshen Song^2^*, Yuxian He^1^*

^1^ NHC Key Laboratory of Systems Biology of Pathogens, National Institute of Pathogen Biology and Center for AIDS Research, Chinese Academy of Medical Sciences and Peking Union Medical College, Beijing 100730, China; ^2^ Research Institute of Youcare Pharmaceutical Group Co., Ltd., Beijing 100176, China; ^3^ Kunming National High-level Biosafety Research Center for Non-Human Primates, Center for Biosafety Mega-Science, Kunming Institute of Zoology, Chinese Academy of Sciences, Kunming, Yunnan 650107, China; ^4^Institute of Hepatology, National Clinical Research Center for Infectious Disease, Shenzhen Third People's Hospital, The Second Affiliated Hospital, School of Medicine, Southern University of Science and Technology, Shenzhen, Guangdong 518112, China.

^#^These authors contributed equally to this work.

^*^To whom correspondence should be addressed. Zheng Zhang, zhangzheng1975@aliyun.com; Minghua Li, [limh@mail.kiz.ac.cn](mailto:limh@mail.kiz.ac.cn); Gengshen Song, songgengshen@youcareyk.com; Yuxian He, yhe@ipb.pumc.edu.cn.

**SUPPLEMENTARY MATERIALS AND METHODS**

***In vitro* cytotoxicity of lipopeptide inhibitors**

Cytotoxicity of lipopeptides was measured using Cell-Counting Kit-8 (CCK-8) (Abbkine, Wuhan, China). In brief, 293T/ACE2, Huh-7 or Vero E6 cells were seeded on a 96-well tissue culture plate (1 × 10^4^ cells per well), and 50 μl volumes of a lipopeptide at different concentrations were added to the cells. After incubation at 37°C for 48 h, 20 μl of CCK-8 solution was added into each well and incubated 2 h at 37°C. Absorbance at 450 nm was then measured using a Multiskan MK3 microplate reader (Thermo Fisher Scientific, Waltham, MA, USA), and percentage of cell viability and median cytotoxic concentration (CC_50_) values were calculated.

**Sensitivity of lipopeptides to** **proteolytic enzymes**

The concentration of a lipopeptide was set at 2 mg/ml in PBS (pH 7.2). Proteinase K, trypsin or chymotrypsin (Sigma-Aldrich) was added with a protease/peptide ratio of 1:20 (wt/wt), followed by incubation at 37°C. Samples were collected at different times (0, 30, 60, 120 and 180 min) and then stored at -20°C before testing. The antiviral activity of each sample was detected by PsV-based single-cycle infection assay.

**Metabolic stability of lipopeptides in liver microsomes**

A phase I metabolic stability kit/human liver microsome (mixed) reagent (iPhase Pharmaceutical Services) was used according to the instruction provided by the manufacturer. Briefly, 10 μL of a solution A and 2 μL of a solution B in the kit were evenly mixed with 28 μL of 0.1 M of a PBS buffer, and the mixture was pre-incubated at 37℃ for 5 min, then sub-packaged in 40 μL/tube, subjected to a warm bath in water bath at 37℃, and prepared into a pre-incubation solution for later use. 154 μL of 0.1 M of a PBS buffer, 5 μL of liver microsomes, and 1 μL of the test lipopeptide solutions at a concentration of 4 mM were mixed, 40 μL of the pre-incubation solution was added, the mixture was immediately incubated in a 37℃-water bath, and timing was conducted. Different incubation time points were set, 200 μL of precooled acetonitrile was added into the incubation system to terminate the reaction, and the antiviral activity of each sample was detected by PsV-based single-cycle infection assay.

**Metabolic stability of** **lipopeptides in human serum**

20% human serum and a test lipopeptide at a final concentration of 150 μM were mixed; the mixture was incubated at 37℃ for 0, 5, 30, 60, 120 or 180 min, and the antiviral activity of each sample was detected by PsV-based single-cycle infection assay.

**Metabolic stability of lipopeptides stored at 37°C**Lipopeptides were stored at 37°C and collected at different time points (3, 7, 14, 21 and 28 days) and then stored at -20°C before testing. The antiviral activity of each sample was detected by PsV-based single-cycle infection assay.

**Pharmacokinetic profiles of IPB29-IS in golden hamsters by inhalation**

The objective of this study was to assess the pharmacokinetic (PK) profile of IPB29 after a single inhalation administration of IPB29-IS. A total of 48 golden hamsters (24/sex) were randomly assigned to 4 groups (6/sex/group) including oronasal low dose, oronasal high dose, induction cartridge low dose and induction cartridge high dose groups. Part of the animals was transferred to oronasal high dose tissue collection group and induction cartridge high dose tissue collection group after 9 days washout period.

Blood samples were collected at pre-dose and 32 min, 45 min, 1 h, 1.5 h, 2.5 h, 4 h, 8 h, 12 h and 24 h after the start of administration from animals in groups 1 to 4 on day 1. Blood and lung samples were collected at pre-dose, 4 hours after the start of administration in group 5 and at pre-dose, 1 h, 4 h, 8 h and12 h after the start of administration in group 6 on day 10.

In the process of inhalation for oronasal groups, the aerosol concentration was sampled and analyzed, and the aerosol particle size distribution was analyzed at the end of administration.

The plasma and lung concentrations of IPB29 were analyzed by LC-MS/MS methods with an LLOQ of 0.5 and 1 ng/mL, respectively. PK parameters were analyzed by non-compartmental analysis (NCA) with WinNonlin 8.0.0.3176 to assess the PK characteristics of IPB29.

**Pharmacokinetic profiles of IPB29-IS in Beagle dogs by intravenous and inhalation**

The objective of this study was to assess the PK profile of IPB29-IS after intravenous (IV) injection or inhalation in Beagle dogs. A total of 24 Beagle dogs (12/sex) were randomly assigned to 4 groups (3/sex/group): intravenous group (1 mg/kg), inhalation groups of low dose (average delivery dose level of 1.566 mg/kg), middle dose (average delivery dose level of 4.885 mg/kg), and high dose (average delivery dose level of 13.303 mg/kg) with a single administration for all groups. Blood samples were collected from animals of the intravenous group at pre-dose, 2 min, 10 min, 0.5 h, 1 h, 2 h, 4 h, 6 h, 8 h, and 24 h after dosing. Blood samples from animals of the low dose inhalation groups were collected at pre-dose, 15 min, 30 min, 1 h, 2 h, 4 h, 6 h, 8 h, 10 h, 12 h and 24 h after the start of administration. Blood samples from animals of the middle dose inhalation groups were collected at pre-dose, 30 min, 1 h, 1.5 h, 2.5 h, 4 h, 6 h, 8 h, 10 h, 12 h and 24 h after the start of administration. Blood samples from animals of the high dose inhalation groups were collected at pre-dose, 45 min, 90 min, 2 h, 3 h, 4 h, 6 h, 8 h, 10 h, 12 h and 24 h after the start of administration.

In the process of inhalation, the aerosol concentration was sampled and analyzed, and the aerosol particle size distribution was analyzed at the end of administration.

The plasma concentrations of IPB29 were analyzed using a validated LC-MS/MS method with an LLOQ of 1 ng/mL. Pharmacokinetics parameters were analyzed using NCA with WinNonlin (8.0.0.3176) to assess the PK characteristics of IPB29.

**Pharmacokinetic and tissue distribution of IPB29-IS in SD rats by intravenous and inhalation**

The objective of this study was to assess the PK profile of IPB29 after IV injection or inhalation administration of IPB29-IS and the lung and trachea tissue distribution of IPB29 after inhalation administration in Sprague-Dawley (SD) rats. A total of 84 SD rats (42/sex) were randomly assigned to 5 groups (18 animals/sex in tissue (lung and trachea) distribution group, 6 animals/sex in remaining groups) including intravenous group (3 mg/kg), inhalation groups of low dose (delivery dose level of 3.896 mg/kg), middle dose (delivery dose level of 11.259 mg/kg), high dose (delivery dose level of 32.401 mg/kg), and tissue distribution group (delivery dose level of 11.259 mg/kg). All animals received a single dose at designated dose level. Blood samples were collected from animals of the intravenous group at pre-dose, 2 min, 5 min, 15 min, 0.5 h, 1 h, 2 h, 4 h, 6 h, 8 h, and 24 h after dosing. Blood samples from animals of the low dose inhalation groups were collected at pre-dose, 52 min, 65 min, 80 min, 2 h, 3 h, 5 h, 7 h, 10 h, 12 h and 24 h after the start of administration. Blood samples from animals of the middle dose inhalation groups were collected at pre-dose, 72 min, 85 min, 100 min, 2 h, 3.5 h, 5.5 h, 8 h, 10 h, 12 h and 24 h after the start of administration. Blood samples from animals of the high dose inhalation groups were collected at pre-dose, 152 min, 165 min, 3 h, 3.5 h, 4.5 h, 6.5 h, 8 h, 10 h, 12 h and 24 h after the start of administration. Blood, lung and trachea tissue samples were collected from animals of tissue distribution group at 2 h, 4 h, 8 h, 24 h, 36 h and 48 h after the start of administration, respectively.

In the process of inhalation, the aerosol concentration was sampled and analyzed, and the aerosol particle size distribution was analyzed at the end of administration.

The concentrations of IPB29 in rat plasma and lung tissue homogenate supernatant were analyzed using two validated LC-MS/MS methods with an LLOQ of 0.5 ng/mL for plasma and 1 ng/mL for lung tissue homogenate supernatant. PK parameters were analyzed using NCA with WinNonlin 8.0.0.3176 to assess the PK and distribution characteristics of IPB29.

**Plasma protein binding of IPB29**

The protein binding of IPB29 in plasma of ICR/CD-1 mouse, SD rat, Beagle dog, cynomolgus monkey and human was evaluated by equilibrium dialysis. The mouse, rat, dog, monkey and human plasma samples with IPB29 concentration of 0.1 µM, 1 µM and 10 µM were added to one side of the dialysis membrane as the test chamber, and the PB solution with pH7.4 (containing 0.002% Tween-80) was added to the other side of the dialysis membrane as the receiving chamber. After incubation at 37℃ for 6 h, the samples were taken from both sides, and detected by LC-MS/MS to calculate the protein binding of IPB29.

**A 7-day repeated inhalation dose range finding study of IPB29-IS in SD rats**

The objectives of this non-GLP study (R22-S111-DR) were to evaluate the possible toxic reactions and metabolism in SD rats after repeated inhalation of 5, 10 and 20 mg/mL IPB29-IS once daily for 7 consecutive days using the Germany PARI compression atomizer system. A total of 60 rats (30 rats/sex) were used in this study, and randomly assigned to 8 groups, which 4 groups (3 rats/sex/group) were used as the main test groups for toxicological study, and 4 groups (3 rats/sex/group for control, 5 rats/sex/group for treated) were used as satellite groups for toxicokinetic analysis. Animals in control groups inhaled clean air as a negative control group, animals in the low dose groups were given 5 mg/mL test article by inhalation for 60 minutes, animals in the mid dose groups were given 10 mg/mL test article by inhalation for 100 minutes, and animals in high dose groups were given the 20 mg/mL test article by inhalation for 360 minutes. Inhalation was performed once daily for 7 consecutive days.

During the study period, main study animals were evaluated for clinical observations (general and detailed clinical observation), body weight, respiratory function (tidal volume, respiratory frequency, and minute volume), hematology, coagulation, clinical chemistry and urinalysis. Blood samples were collected on day 1 and day 7 for determination of drug concentration and toxicokinetic analysis and on day 8 for anti-drug antibodies analysis. At the end of the administration period (day 8), lung weights were measured and main study animals were examined by gross anatomy and histopathology of respiratory tract related organs (nasal cavity, olfactory bulb, larynx, trachea, lungs, and tongue) was performed.

IPB29 concentration analysis was performed during administration from day 1 to day 7, and the aerosol particle size distribution was analyzed after the dosing on day 1 and day 7. The actual delivered dose was 4.07 ± 0.44 mg/kg for the low-dose group, 12.04 ± 1.06 mg/kg for the mid-dose group, and 72.46 ± 6.56 mg/kg for the high-dose group. The T_99_ (min) of the exposure system was approximately 1.64 min (T_99_ = 4.6 × V/a (V = 0.00321 m^3^, a = 0.009 m^3^/min)). For the high dose, the mass median aerodynamic diameter (MMAD) was 3.275 μm; the geometric standard deviation (GSD) was 1.752, and the fine particle fraction (FPF, Fine Particle Fraction, ˂ 5μm) was 77.738%.

**A 4-week toxicity Study of IPB29-IS administered by inhalation in SD rat with a 4-week recovery period**

The objectives of this study (R22-S111-RD) were to evaluate potential toxicity and the toxicokinetic profile of IPB29-IS with different delivery doses administered by inhalation to SD rats once daily for 4 consecutive weeks and the reversibility of toxicity following a 4-week recovery period. A total of 192 SD rats (96/sex) were randomly assigned into 8 groups. Four groups (15 animals/sex/group) were used for the main toxicity study. Four satellite groups (4/sex for controls, 8/sex for treated groups; plus 2/sex/group as spares) were dosed for toxicokinetic analysis. Animals in control groups were given clean air as the negative control group, animals in the low, mid and high groups were given different concentrations (5, 10 and 20 mg/mL) of IPB29-IS by inhalation using a PARI nebulizer. Dosing was once daily for 28 consecutive days; the duration of inhalation was 90, 150 and 240 minutes for the low, medium and high dose groups, respectively. The inhalation time for the negative control group was 240 minutes.

Animals in the main study were evaluated for clinical observations (daily clinical observations, detailed clinical observation and site observation after dosing), body weight, food consumption, body temperature, ophthalmoscopic examinations, hematology, coagulation, clinical chemistry, urinalysis, immunophenotyping (CD3^+^, CD3^+^CD4^+^, CD3^+^CD8^+^, CD3^+^CD4^+^/CD3^+^CD8^+^), anti-drug antibody and cytokine assays (TNF-α, IFN-γ, IL-2, IL-4, IL-6 and IL-12). Blood samples were collected on day 1 and day 28 for blood drug concentration and toxicokinetic analysis. The first 10 animals/sex in main study were euthanized at the end of 4-week dosing period (day 29), the remaining animals were euthanized at the end of recovery period (day 57). Organ weights, gross and microscopic evaluations were performed.

During the study, the aerosol concentration was analyzed during the dosing from day 1 to day 28. On days 1, 7, 14, 21, 26 and 28, samples were collected after the end of dosing for aerosol particle size measurement. The actual delivered dose was 5.264 ± 0.695 mg/kg in the low dose group, 19.246 ± 2.222 mg/kg in the medium dose group and 49.570 ± 5.205 mg/kg in the high dose group. The particle size distribution data showed that the MMAD was between 2.2 to 3.5 μm (average GSD 1.738 – 2.008) and the FPF was between 74.534% to 88.942%. T_99_ is about 1.48 to 1.64 min.

**A 7-day repeated inhalation dose range finding study of IPB29-IS in Beagle dogs**

The objectives of this non-GLP (D22-S111-DR) study were to evaluate the toxicity in Beagle dogs after repeated inhalation of IPB29-IS at 5, 10, and 20 mg/mL once daily for 7 consecutive days, a total of 12 dogs (6/sex) were randomly assigned into 4 groups (3/group, two males/ one female or one male/two females). Animals in the control group were given clean air as the negative control group; animals in the low, mid and high groups were given 5, 10 or 20 mg/mL of IPB29-IS. The duration of inhalation was 120, 60, 90 and 120 minutes for the control, low, medium and high dose groups, respectively. Inhalation was once daily for 7 consecutive days using a Germany PARI compression atomizer.

Animals were evaluated for clinical observations (daily clinical observations and detailed clinical observation), body weight, body temperature, electrocardiogram (limb lead II ECG), respiratory function (respiratory rate, tidal volume), hematology, coagulation, clinical chemistry, urinalysis, anti-drug antibody and toxicokinetic analysis. Animals were euthanized and necropsied at the end of the dosing period (day 8) and histopathological examination of major respiratory organs (nasal cavity, olfactory bulb, larynx, trachea, lung, tongue) was performed.

The aerosol concentration was analyzed during the dosing from day 1 to day 7. On day 1 and day 7, samples of high-dose group were collected after dosing for aerosol particle size measurement. The actual delivered dose was 2.154 ± 0.208 mg/kg in the low dose group, 5.800 ± 0.655 mg/kg in the mid dose group and 13.269 ± 2.860 mg/kg in the high dose group. The T_99_ (min) of the exposure system was 0.018 min (T_99_ = 4.6 × V/a (V = 0.003 m^3^, a = 0.767 m^3^/min)). For the high dose, the MMAD was 2.874 μm, the GSD was 1.842, and the FPF was 81.473%.

**A 4-week toxicity study of IPB29-IS administered by inhalation in Beagle dogs with a 4-week recovery period**

The objectives of this study (D22-S111-RD) were to evaluate potential toxicity and the toxicokinetic profile of IPB29-IS with different delivery doses administered by inhalation to Beagle dogs once daily for 4 consecutive weeks and the reversibility of toxicity following a 4-week recovery period. A total of 40 dogs (20/sex) were randomly assigned into 4 groups (5/sex/group). Animals in the control were given clean air as a negative control group for 120 minutes; animals in the low, mid and high dose groups were given 5, 10, or 20 mg/mL of IPB29-IS for 60, 90, and 120 minutes, respectively. Dosing was once daily for 4 weeks (total of 28 doses) using German PARI nebulizers. The first 3 animals/sex in each group were euthanized at the end of the dosing period (day 29), and the remaining 2 animals/sex/group were euthanized on day 57 after the end of a 4-week recovery period. All animals underwent complete gross observation and histopathological examination.

Animals were observed for the death/moribundity and clinical symptoms. Body weight, food consumption, body temperature, blood pressure, safety pharmacology tests (respiratory parameters [Jacket telemetry]), ECG (Limb Ⅱ)), ophthalmic examination, clinical pathology (hematology, coagulation, clinical chemistry and urinalysis), immunophenotyping (CD3^+^, CD3^+^CD4^+^, CD3^+^CD8^+^, CD3^+^CD4^+^/CD3^+^CD8^+^), anti-drug antibodies, and toxicokinetics were monitored periodically.

During the study, the aerosol concentration was analyzed during the dosing from day 1 to day 28. On days 1, 7, 14, 21 and 28, samples were collected after the end of dosing for aerosol particle size measurement. The actual delivered dose was 2.475 ± 0.358 mg/kg in the low dose group, 5.568 ± 0.973 mg/kg in the mid dose group and 14.798 ±2 .430 mg/kg in the high dose group. The particle size distribution data showed that the MMAD was between 2.3 to 3.5 μm (GSD was 1.785 -1.1912) and the FPF was between 61.635 % to 88.789 %. T_99_ is about 0.69 to 0.77 min.

**Mutagenicity test of IPB29-IS in *Salmonella Typhimurium***

The objective of this study was to evaluate the potential mutagenicity of IPB29-IS in *Salmonella Typhimurium*. The mutagenicity of test article was evaluated using histidine deficient *Salmonella Typhimurium* tester strains TA97a, TA98, TA100, TA102, and TA1535. According to ICH guideline and the preliminary results, five concentrations (10, 30, 100, 300, and 800 μg/plate) were selected for testing in the TA97a, TA98, TA100, TA102 and TA1535 strains. A spontaneous control group was included. The positive controls of direct mutagens 9-Aminoacridine (9-AA), 2-Nitrofluorene (2-NF), N4-Aminocytidine (N4-ACT) and 4-Nitroquinoline N-oxide (4-NQO), and the indirect mutagen 2-Aminoanthracene (2-AA) were also included in the tests. After incubation for about 65 hours at 37ºC with S9 metabolic activation (+S9) or without S9 metabolic activation (-S9), revertant colonies were counted on all plates. Background lawn was also examined microscopically. Each treatment consisted of triplicate plates.

Precipitation was observed during the process of dosing at concentrations ≥ 300 µg/plate with or without S9 (±S9) for the TA97a, TA98, TA100, TA102 and TA1535 strains.

**Chromosomal aberration study of IPB29-IS in Chinese hamster lung fibroblasts**

The objective of this study was to evaluate the potential genotoxicity of IPB29-IS, by testing the induction of chromosomal aberration in Chinese hamster lung (CHL) fibroblast cells with or without a rat liver S9 metabolic activation system. Based on the preliminary test results and ICH guideline S2 (R1), concentrations of 8, 15, 30 and 45 μg/mL were selected for the 4-hour treatment with and without metabolic activation (±S9); concentrations of 4, 8, 15 and 20 μg/mL were selected for the 24-hour treatment without metabolic activation (-S9). Vehicle control (sterile water for injection) was included. Positive control groups were also included with mitomycin C (MMC) at 0.2 µg/mL as a direct clastogen, and cyclophosphamide monohydrate (CP) at 10 µg/mL as an indirect clastogen.

The CHL cells were cultured with test or control article for 24 hours without S9, or for 4 hours with or without S9 followed by addition of fresh normal culture medium without test or control article for 20 hours. Cell cultures were treated with colchicine (0.01 mg/mL, 0.1 mL) for 4 hours prior to harvesting cells for examination. At the end of incubation, the cells of each flask were harvested and counted, then treated with hypotonic solution (0.075 mol/L KCl). After fixed in fixative solution and stained with Giemsa solution, the cells were examined microscopically for chromosome evaluation. 300 well-spread metaphase cells were evaluated for treatments with the test article and vehicle. One hundred (100) well-spread metaphase cells were examined for treatments with MMC and CP with S9. The chromosomal aberration rate for each treatment was calculated.

**Micronucleus study of IPB29-IS in bone marrow polychromatic erythrocytes of CD-1 mice by intravenous injection**

The objective of this study was to evaluate the potential genotoxicity of IPB29-IS using the micronucleus test, after administration to CD-1 mice by intravenous injection at 3, 10 and 35 mg/kg for 2 days consecutively. 32 male mice (6/group except 8/group for the high dose) were administered vehicle control (sterile water for injection) or test article (3, 10 and 35 mg/kg) by intravenous injection once a day for 2 consecutive days. Animals in the positive control article group (CP at 60 mg/kg) were dosed via intraperitoneal injection once on day 2. The dose volume for all groups was 10 mL/kg. Bone marrow smears were performed on all surviving animals about 22 hours after the final dose. The bone marrow smears of the first 6 surviving animals according to the order of animal number in 35 mg/kg dose group and all the surviving animals in the other groups were observed. The micronucleus numbers from 4000 PCE (polychromatic erythrocytes) were counted, and 500 erythrocytes (PCE+NCE (normochromatic erythrocytes)) were counted to determine the ratio of PCE/(PCE+NCE).

**Active systemic anaphylaxis study of IPB29-IS administered by inhalation in** **guinea pigs**

The objective of this study was to evaluate the active anaphylactic reaction of IPB29-IS administered by inhalation in guinea pigs. A total of 36 male hartley guinea pigs were randomly assigned to 4 groups (9/group). Clean air was administered to animals in the control group (negative control) for 60 min of inhalation sensitization, and 120 min of inhalation challenge. Liquid aerosol generated from albumin from chicken egg white at 20 mg/mL was delivered to animals in the positive control group for 5 minutes of sensitization, and 10 min of challenge. IPB29-IS (10 mg/mL) was administered by Germany PARI compression atomizer to animals in the low-dose group for 20 min of inhalation sensitization and 40 min of inhalation challenge. The IPB29-IS (10 mg/mL) was administered to animals in the high-dose group for 60 min of inhalation sensitization and 120 min of inhalation challenge. After sensitization dosing on day 1, day 3 and day 5, the first 3 animals in every group were challenged on day 19. The animals in test article groups showed no anaphylactic symptoms after the day 19 challenge, the remaining animals in each group were challenged on day 26.

During the inhalation of sensitization dosing and challenge dosing, aerosol samples were collected and analyzed for concentration. At the end of administration on day 1, day 5 and day 19, aerosols were sampled and analyzed for aerosol particle size distribution. The actual average sensitizing delivery dose, average challenge delivery dose and aerosol particle size distribution parameters are shown in Table S1.

**SUPPLEMENTARY FIGURES**


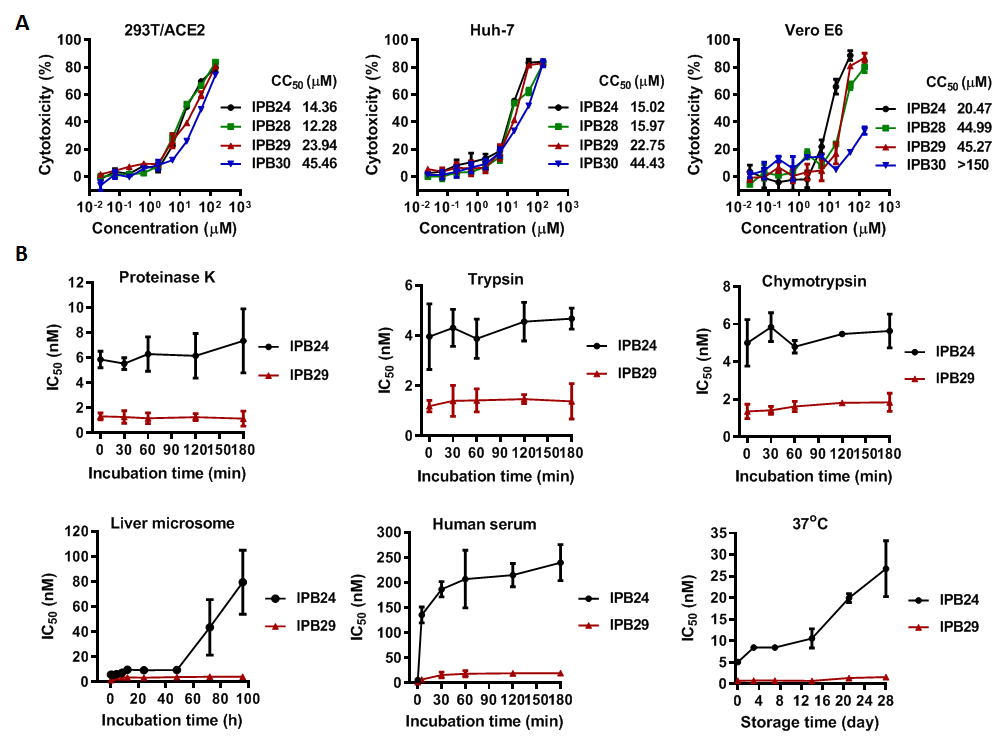


**Figure S1 The *in vitro* cytotoxicity and stability of lipopeptides.** (**A**) The cytotoxicity of lipopeptides on 293T/ACE2, Huh-7, and Vero E6 cells was determined at different concentrations by Cell-Counting Kit-8. (**B**) The stability of lipopeptides treated with different proteolytic enzymes, human liver microsome, human serum, and temperature.


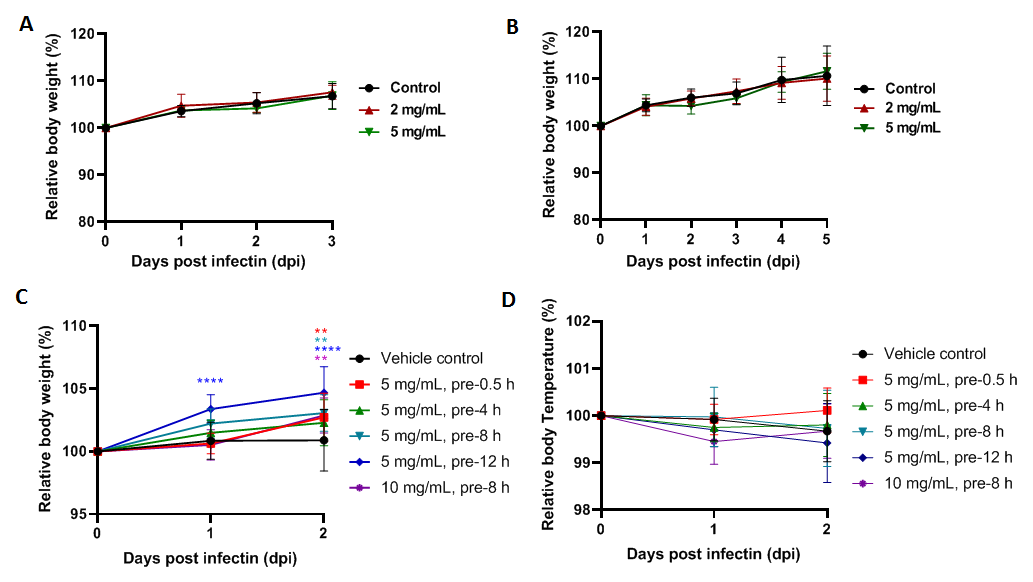


**Figure S2 The body weights and temperature and of IPB29-IS treated hamsters.** (**A-B**) The body weights of hamsters treated with IPB29-IS for 3 days (**A**) or 5 days (**B**). The body weights (**C**) and temperature (**D**) of hamsters pre-treated with IPB29-IS at different time points.

**SUPPLEMENTARY TABLES**

**Table S1. The dosing groups of the study**

| Group | | Conc.  (mg/mL) | Inhalation  Time  (min) | Dose Route |
| --- | --- | --- | --- | --- |
|  |  |  |  |  |
| 1 | Oronasal Low Dose | 2 | 30 | Oronasal aerosol inhalation |
| 2 | Oronasal High Dose | 5 | 30 | Oronasal aerosol inhalation |
| 3 | Induction Cartridge Low Dose | 2 | 30 | Induction Cartridge Atomization Inhalation |
| 4 | Induction Cartridge High Dose | 5 | 30 | Induction Cartridge Atomization Inhalation |
| Wash-out Period (9 days) | | | | |
| 5 | Oronasal High Dose  Tissue Collection | 5 | 30 | Oronasal aerosol inhalation |
| 6 | Induction Cartridge High Dose  Tissue Collection | 5 | 30 | Induction Cartridge Atomization Inhalation |

**Table S2. The actual delivered dose and aerosol size distribution parameters**

| Items | Group 1 | Group 2 | Group 5 |
| --- | --- | --- | --- |
| Delivered dose (mg/kg) | 0.854 | 2.432 | 2.024 |
| MMAD (μm) | 2.703 | 3.072 | 3.312 |
| GSD | 1.728 | 1.783 | 1.904 |
| FPF (%) | 87.155 | 80.04 | 74.032 |

Note: MMAD: Mass Median Aerodynamic Diameter; GSD: Geometric Standard Deviation; FPF: Fine Particle Fraction (MMAD < 5 μm).

**Table S3. The Mean PK Parameters of IPB29 after a single inhalation of IPB29-IS with average delivery doses of 0.854 mg/kg, 2.432 mg/kg for oronasal low and high dose groups**

| Group | Dose Level | Sex | t_1/2_ | T_max_ | C_max_ | AUC_last_ | AUC_inf_ | V_d_ | Cl | MRT_last_ |
| --- | --- | --- | --- | --- | --- | --- | --- | --- | --- | --- |
|  | mg/kg |  | h | h | ng/mL | h·ng /mL | h·ng /mL | L/kg | L/h/kg | h |
| 1  Oronasal Low Dose | 0.854 | Male | 4.40 | 2.50 | 5.29 | 37.88 | 46.47 | 116.65 | 18.38 | 5.37 |
|  |  | Female | 4.45 | 2.50 | 5.22 | 39.88 | 48.91 | 112.05 | 17.46 | 5.28 |
| 2  Oronasal High Dose | 2.432 | Male | 6.80 | 0.75 | 22.89 | 74.86 | 112.13 | 212.73 | 21.69 | 5.26 |
|  |  | Female | 5.55 | 0.75 | 7.05 | 54.68 | 74.92 | 260.11 | 32.46 | 5.67 |

**Table S4. The mean PK parameters of IPB29 after a single inhalation of IPB29-IS for induction cartridge low and high dose groups**

| Group | Dose  Concentration | Sex | t_1/2_ | T_max_ | C_max_ | AUC_last_ | AUC_inf_ | V_d_ | Cl | MRT_last_ |  |
| --- | --- | --- | --- | --- | --- | --- | --- | --- | --- | --- | --- |
|  | mg/mL |  | h | h | ng/mL | h·ng /mL | h·ng /mL | L/kg | L/h/kg | h |  |
| 3  Induction Cartridge  Low Dose | 2 | Male | NA | 4.00 | 6.47 | 52.91 | NA | NA | NA | 5.60 |  |
|  |  | Female | 5.87 | 2.50 | 6.80 | 63.22 | 68.13 | 248.48 | 29.36 | 7.91 |  |
| 4  Induction Cartridge  High Dose | 5 | Male | 6.23 | 1.00 | 62.50 | 132.90 | 144.86 | 310.32 | 34.52 | 7.91 |  |
|  |  | Female | 4.84 | 1.00 | 18.98 | 136.67 | 142.12 | 245.69 | 35.18 | 7.97 |  |

Note: “NA” = Not Applicable.

**Table S5. The mean plasma and lung concentrations of IPB29 after a single inhalation of IPB29-IS with average delivery dose of 2.024 mg/kg for oronasal high dose tissue collection Group**

| Group | Dose Level | Sex | Index | Lung concentration  (ng/g) | | Plasma concentration  (ng/mL) | | Lung/Plasma 4h |
| --- | --- | --- | --- | --- | --- | --- | --- | --- |
|  | mg/kg |  |  | Pre-dose | 4h | Pre-dose | 4h | concentration ratio |
| 5  Oronasal High Dose  Tissue Collection | 2.024 | Male | Mean | 0.00 | 4486.44 | 0.00 | 10.04 | 446.86 |
|  |  |  | SD | 0.00 | 401.93 | 0.00 | 1.94 |  |
|  |  | Female | Mean | 0.00 | 2958.32 | 0.00 | 8.27 | 357.72 |
|  |  |  | SD | 0.00 | 521.02 | 0.00 | 2.60 |  |

Note: Plasma density was assigned as 1 g/mL to calculate concentration ratios.

**Table S6. The exposure (calculated by AUC) of IPB29 and the ratio of exposure of IPB29 in lung tissue to plasma**

| Group | Dose  Concentration  mg/mL | Matrix | AUC_last_ (h·ng/g) | | AUC_last_ Ratio | |
| --- | --- | --- | --- | --- | --- | --- |
|  |  |  | Sex | | Lung/Plasma | |
|  |  |  | Male | Female | Male | Female |
| 6 | 5 | Plasma | 119.99 | 129.43 | 550.82 | 409.40 |
| Induction Cartridge High Dose Tissue Collection |  | Lung | 66092.48 | 52988.08 |  |  |

Note: Plasma density was assigned as 1 g/mL to calculate AUC ratios.

**Table S7. The actual delivered dose and aerosol size distribution parameters**

| Items | Low dose group | Middle dose group | High dose group |
| --- | --- | --- | --- |
| Delivered dose (mg/kg) | 1.566 | 4.885 | 13.303 |
| MMAD (μm) | 3.130 | 2.920 | 3.232 |
| GSD | 1.855 | 1.811 | 1.846 |
| FPF (%) | 77.491 | 81.601 | 76.299 |

Note: MMAD: Mass Median Aerodynamic Diameter; GSD: Geometric Standard Deviation; FPF: Fine Particle Fraction (MMAD < 5 μm).

**Table S8. The mean PK parameters of IPB29 in dogs after a single intravenous injection of 1 mg/kg**

| Dose Level | Sex | Index | t_1/2_ | C_0_ | AUC_last_ | AUC_inf_ | V_z_ | Cl | MRT_last_ |
| --- | --- | --- | --- | --- | --- | --- | --- | --- | --- |
| (mg/kg) |  |  | (h) | (ng/mL) | (h·ng/mL) | (h·ng/mL) | (mL/kg) | (mL/h/kg) | (h) |
| 1 | Male | Mean | 7.99 | 17497.20 | 130546.07 | 148880.40 | 79.30 | 6.93 | 7.87 |
|  |  | SD | 0.81 | 4408.31 | 26641.42 | 32876.33 | 15.37 | 1.43 | 0.20 |
|  | Female | Mean | 7.39 | 16922.13 | 127825.42 | 144592.74 | 76.14 | 7.46 | 7.56 |
|  |  | SD | 1.62 | 3139.10 | 38491.32 | 52708.95 | 11.44 | 2.25 | 0.83 |

**Table S9. The mean PK parameters of IPB29 in dogs after a single inhalation with average delivery doses of 1.566 mg/kg, 4.885 mg/kg and 13.303 mg/kg**

| Average delivery Dose | Sex | Index | t_1/2_ | T_max_ | C_max_ | AUC_last_ | AUC_inf_ | V_z_/F | Cl/F | MRT_last_ |
| --- | --- | --- | --- | --- | --- | --- | --- | --- | --- | --- |
| (mg/kg) |  |  | (h) | (h) | (ng/mL) | (h·ng/mL) | (h·ng/mL) | (L/kg) | (L/h/kg) | (h) |
| 1.566 | Male | Mean | 26.10 | 8.67 | 23.83 | 401.58 | 957.32 | 98.52 | 4.74 | 12.96 |
|  |  | SD | 20.42 | 1.15 | 13.69 | 237.57 | 1095.54 | 64.54 | 5.42 | 1.21 |
|  | Female | Mean | 14.67 | 8.00 | 28.19 | 457.47 | 925.65 | 35.48 | 1.86 | 12.45 |
|  |  | SD | 6.80 | 2.00 | 11.26 | 172.65 | 391.01 | 1.60 | 0.78 | 0.79 |
| 4.885 | Male | Mean | 12.02 | 8.00 | 60.42 | 946.76 | 1218.06 | 69.47 | 4.01 | 11.82 |
|  |  | SD | 1.46 | 2.00 | 16.34 | 195.50 | 43.50 | 5.95 | 0.14 | 0.13 |
|  | Female | Mean | 10.91 | 8.00 | 47.61 | 698.28 | 1340.36 | 64.00 | 4.39 | 12.04 |
|  |  | SD | 2.79 | 2.00 | 34.73 | 520.53 | 782.26 | 22.69 | 2.56 | 1.00 |
| 13.303 | Male | Mean | 13.73 | 7.33 | 198.66 | 2960.35 | 3581.03 | 92.53 | 4.16 | 11.90 |
|  |  | SD | 7.31 | 2.31 | 105.92 | 1441.67 | 1654.62 | 81.95 | 1.92 | 0.86 |
|  | Female | Mean | 5.68 | 8.00 | 237.10 | 2974.83 | 3193.75 | 35.04 | 4.40 | 10.59 |
|  |  | SD | 1.00 | 2.00 | 56.25 | 616.77 | 1045.78 | 5.49 | 1.44 | 0.77 |

**Table S10. The PK dose proportionality in dogs following inhalation**

| Analyte | Sex | Dose  (mg/kg) | Dose  Ratio | Mean C_max_  Ratio | Mean AUC_last_  Ratio |
| --- | --- | --- | --- | --- | --- |
| IPB29 | Male | 1.566 | 1.00 | 1.00 | 1.00 |
|  |  | 4.885 | 3.12 | 2.54 | 2.36 |
|  |  | 13.303 | 8.49 | 8.34 | 7.37 |
|  | Female | 1.566 | 1.00 | 1.00 | 1.00 |
|  |  | 4.885 | 3.12 | 1.69 | 1.53 |
|  |  | 13.303 | 8.49 | 8.41 | 6.50 |

**Table S11. The PK dose proportionality in dogs following intravenous administration**

| Analyte | Route | Dose  (mg/kg) | Female/Male Ratio | |
| --- | --- | --- | --- | --- |
|  |  |  | C_max_ | AUC_last_ |
| IPB29 | Intravenous Injection | 1 | 0.98 | 0.98 |
|  | Inhalation | 1.566 | 1.18 | 1.14 |
|  |  | 4.885 | 0.79 | 0.74 |
|  |  | 13.303 | 1.19 | 1.00 |

**Table S12. The inhalation bioavailability in dogs**

| Analyte | Sex | Group | F (%) | |
| --- | --- | --- | --- | --- |
|  |  |  | based on AUC_last_ | based on AUC_inf_ |
| IPB29 | Male | Low dose group (1.566 mg/kg) | 0.20 | 0.41 |
|  |  | Middle dose group (4.885 mg/kg) | 0.15 | 0.17 |
|  |  | High dose group (13.303 mg/kg) | 0.17 | 0.18 |
|  | Female | Low dose group (1.566 mg/kg) | 0.23 | 0.41 |
|  |  | Middle dose group (4.885 mg/kg) | 0.11 | 0.19 |
|  |  | High dose group (13.303 mg/kg) | 0.17 | 0.17 |

**Table S13. The actual delivered dose and aerosol size distribution parameters**

| Items | Low dose group | Middle dose/tissue distribution group | High dose group |
| --- | --- | --- | --- |
| Delivered dose (mg/kg) | 3.896 | 11.259 | 32.401 |
| MMAD (μm) | 3.011 | 3.295 | 4.323 |
| GSD | 1.829 | 1.783 | 1.668 |
| FPF (%) | 79.837 | 76.819 | 62.387 |

Note: MMAD: Mass Median Aerodynamic Diameter; GSD: Geometric Standard Deviation; FPF: Fine Particle Fraction (MMAD < 5 μm).

**Table S14. The mean PK parameters of IPB29 after a single IV injection of 3 mg/kg**

| Group | Dose | Sex | t_1/2_ | C_0_ | AUC_last_ | AUC_inf_ | V_z_ | Cl | MRT_last_ |
| --- | --- | --- | --- | --- | --- | --- | --- | --- | --- |
|  | mg/kg |  | h | ng/mL | h·ng /mL | h·ng /mL | mL/kg | mL/h/kg | h |
| IV Group | 3 | Male | 2.27 | 64504.72 | 123399.00 | 123459.35 | 79.61 | 24.30 | 2.54 |
|  |  | Female | 3.50 | 41816.37 | 72933.49 | 73194.80 | 206.95 | 40.99 | 2.75 |

**Table S15. The mean PK parameters of IPB29 after a single inhalation**

| Group | Delivery  Dose | Index | t_1/2_ | T_max_ | C_max_ | AUC_last_ | AUC_inf_ | V_z_/F | Cl/F | MRT_last_ |
| --- | --- | --- | --- | --- | --- | --- | --- | --- | --- | --- |
|  |  |  | h | h | ng/mL | h·ng /mL | h·ng /mL | L/kg | L/h/kg | h |
| Low Dose | 3.896 | Male | 2.54 | 3.00 | 4.77 | 25.95 | 28.49 | 500.89 | 136.77 | 5.38 |
|  |  | Female | 3.63 | 3.00 | 3.54 | 23.95 | 27.96 | 730.32 | 139.34 | 5.41 |
| Middle Dose | 11.259 | Male | 6.61 | 3.50 | 9.42 | 75.08 | 81.52 | 1317.10 | 138.12 | 7.49 |
|  |  | Female | 8.07 | 3.50 | 10.89 | 76.31 | 84.98 | 1542.89 | 132.49 | 7.86 |
| High Dose | 32.401 | Male | 3.72 | 4.50 | 53.21 | 320.67 | 325.12 | 534.65 | 99.66 | 6.97 |
|  |  | Female | 4.41 | 4.50 | 31.29 | 186.14 | 192.76 | 1068.23 | 168.09 | 7.28 |

**Table S16. Plasma, lung and trachea exposure after inhalation**

| Matrix | AUC_last_ (h·ng/g) | | AUC_last_ Ratio | | |
| --- | --- | --- | --- | --- | --- |
|  | Sex | | Tissue/Plasma | | Female/Male |
|  | Male | Female | Male | Female |  |
| Plasma | 46.96 | 54.79 | 1.00 | 1.00 | 1.17 |
| Lung | 155374.01 | 198016.44 | 3308.35 | 3614.25 | 1.27 |
| Trachea | 52751.01 | 61815.03 | 1123.22 | 1128.26 | 1.17 |

Note: Plasma density was assigned as 1 g/mL to calculate AUC ratios.

**Table S17. Plasma protein binding of IPB29 in plasma of various species**

| Species | Concentration (μM) | Average *f*_b_ (%) |
| --- | --- | --- |
| ICR/CD-1 mouse | 0.1 | 99.97 |
|  | 1 | 99.99 |
|  | 10 | 99.99 |
| SD rat | 0.1 | 99.91 |
|  | 1 | 99.99 |
|  | 10 | 100.00 |
| Beagle dog | 0.1 | 99.98 |
|  | 1 | 99.99 |
|  | 10 | 100.00 |
| Cynomolgus monkey | 0.1 | 99.98 |
|  | 1 | 100.00 |
|  | 10 | 100.00 |
| Human | 0.1 | 99.96 |
|  | 1 | 100.00 |
|  | 10 | 100.00 |

**Table S18. Metabolic Stability of IPB29 in Plasma and Hepatocytes**

| Name | Species | The Remaining Rate for the Parent Drug in Plasma | The Remaining Rate for the Parent Drug in Hepatocytes |
| --- | --- | --- | --- |
| IPB29 | ICR/CD-1 mouse | 104% | 61.5% |
|  | SD rat | 109% | 64.4% |
|  | Beagle dog | 109% | 7.04% |
|  | Cynomolgus monkey | 107% | 50.7% |
|  | Human | 109% | 43.1% |

**Table S19. IPB29-IS toxicology program**

| Study Type and Duration | Route of Administration | Species | GLP^a^ |  |
| --- | --- | --- | --- | --- |
| Repeat-Dose Toxicity | | | | |
| 7-Day repeat-dose toxicity study | Inhalation | Rat | No |  |
| 4-Week repeat-dose toxicity study with 4-Week recovery | Inhalation | Rat | Yes |  |
| 7-Day repeat-dose toxicity study | Inhalation | Dog | No |  |
| 4-Week repeat-dose toxicity study with 4-Week recovery | Inhalation | Dog | Yes |  |
| Genotoxicity | | | | |
| *In vitro* reverse mutation assay | *In vitro* | *Salmonella typhimurium* | Yes |  |
| *In vitro* chromosomal aberration assay | *In vitro* | Chinese Hamster lung fibroblasts | Yes |  |
| *In vivo* micronucleus assay | Intravenous | Mouse | Yes |  |
| Local Tolerance | | | |  |
| Active systemic anaphylaxis | Inhalation | Guinea Pig | Yes |  |

Abbreviation: GLP=Good Laboratory Practice

^a^An entry of “Yes” indicates that the study includes a GLP-compliance statement.

**Table S20. Toxicokinetic parameters for IPB29 in rats (7-day repeat-dose toxicity study)**

| Dose (mg/kg/day) | C_max_  ng/mL | | AUC_last_  ng•h/mL | |
| --- | --- | --- | --- | --- |
|  | Day 1 | Day 7 | Day 1 | Day 7 |
| *Males* |  |  |  |  |
| 4.07 | 5.27 | 43.73 | 15.81 | 46.24 |
| 12.04 | 31.25 | 46.42 | 74.13 | 105.70 |
| 72.46 | 309.71* | 182.18 | 1539.66* | 624.43 |
| *Females* |  |  |  |  |
| 4.07 | 4.81 | 15.73 | 23.69 | 40.13 |
| 12.04 | 15.87 | 63.46 | 61.14 | 106.09 |
| 72.46 | 234.98* | 301.25 | 876.48* | 866.98 |

Values for animal numbers 2231481, 2231488 were not included in the calculated parameters

**Table S21. Toxicokinetic parameters for IPB29 in rats (4-week repeat-dose toxicity study)**

| Average Delivered Dose (mg/kg/day) | C_max_  ng/mL | | AUC_last_  ng•h/mL | |
| --- | --- | --- | --- | --- |
|  | Day 1 | Day 28 | Day 1 | Day 28 |
| *Males* |  |  |  |  |
| 5.264 | 5.77 | 5.09 | 33.54 | 59.87 |
| 19.246 | 27.25 | 25.12 | 237.32 | 191.20 |
| 49.570 | 106.38 | 39.60 | 680.18 | 273.11 |
| *Females* |  |  |  |  |
| 5.264 | 4.66 | 3.19 | 33.57 | 38.23 |
| 19.246 | 21.22 | 18.46 | 189.53 | 166.57 |
| 49.570 | 69.01 | 38.25 | 649.62 | 343.77 |

Table S22. Toxicokinetic parameters for IPB29 in dogs (7-day repeat-dose toxicity study)

| Actual Delivered Dose (mg/kg/day) | C_max_  ng/mL | | AUC_last_  ng•h/mL | |
| --- | --- | --- | --- | --- |
|  | Day 1 | Day 7 | Day 1 | Day 7 |
| *Combined Sex* |  |  |  |  |
| 2.154 | 14.20 | 14.37 | 219.71 | 269.40 |
| 5.800 | 25.75 | 56.69 | 437.53 | 825.01 |
| 13.269 | 61.38 | 125.25 | 961.36 | 2002.55 |

Table S23. Toxicokinetic parameters for IPB29 in dogs (4-week repeat-dose toxicity study)

| Dose (mg/kg/day) | C_max_  ng/mL | | AUC_0-24h_  ng•h/mL | |
| --- | --- | --- | --- | --- |
|  | Day 1 | Day 28 | Day 1 | Day 28 |
| *Males* |  |  |  |  |
| 2.475 | 18.36 | 14.82 | 291.63 | 257.90 |
| 5.568 | 63.38 | 29.21 | 907.57 | 411.46 |
| 14.798 | 250.16 | 84.34 | 3422.56 | 1186.88 |
| *Females* |  |  |  |  |
| 2.475 | 20.01 | 16.59 | 304.80 | 255.42 |
| 5.568 | 33.57 | 63.27 | 490.80 | 1130.65 |
| 14.798 | 209.37 | 82.09 | 2704.16 | 1255.30 |

Table S24. Average activation rate or average inhibition rate of IPB29 against 6 types of pharmacological targets

| Serial  number | Family | Target | IPB29（10 μM） | |
| --- | --- | --- | --- | --- |
|  |  |  | Average activation rate (%) | Average inhibition rate (%) |
| GPCR class | | | | |
| 1 | Adenosine | A_2A_ | 0.47 | -1.48 |
| 2 | Adrenergic | α_1A_ | -3.76 | 9.07 |
| 3 | Adrenergic | α_2A_ | -0.47 | 20.06 |
| 4 | Adrenergic | β_1_ | -3.00 | 8.98 |
| 5 | Adrenergic | β_2_ | -2.26 | 14.28 |
| 6 | Cannabinoid | CB_1_ | 13.06 | -16.43 |
| 7 | Cannabinoid | CB_2_ | 34.39 | -16.59 |
| 8 | Cholecystokinin | CCK_1_ | -0.65 | 4.41 |
| 9 | Dopamine | D_1_ | 0.37 | -0.06 |
| 10 | Dopamine | D_2S_ | -3.54 | 4.52 |
| 11 | Endothelin | ET_A_ | 5.55 | -16.19 |
| 12 | Histamine | H_1_ | -1.08 | 0.65 |
| 13 | Histamine | H_2_ | 5.15 | -6.96 |
| 14 | Acetylcholine | M_1_ | -5.54 | 6.69 |
| 15 | Acetylcholine | M_2_ | -1.89 | 15.05 |
| 16 | Acetylcholine | M_3_ | -1.51 | 6.16 |
| 17 | Opioid | Delta | 0.04 | 8.89 |
| 18 | Opioid | Kappa | -2.40 | 32.91 |
| 19 | Opioid | Mu | -0.48 | 12.66 |
| 20 | 5-Hydroxytryptamine | 5HT_1A_ | -0.67 | 14.87 |
| 21 | 5-Hydroxytryptamine | 5HT_1B_ | -0.77 | -3.09 |
| 22 | 5-Hydroxytryptamine | 5HT_2A_ | -1.27 | 12.66 |
| 23 | 5-Hydroxytryptamine | 5HT_2B_ | -5.08 | 6.43 |
| 24 | Vasopressin | V_1A_ | -1.60 | 32.88 |
| Ion channel class | | | | |
| 25 | Calcium channel | Cav1.2 | NA | 4.76 |
| 26 | Sodium channel | Nav1.5 | NA | 41.54 |
| 27 | Potassium channel | hERG | NA | 75.20 |
| 28 | Potassium channel | KvLQT1(7.1)/mink | NA | 15.57 |
| 29 | Potassium channel | Kir2.1 | NA | 0.86 |
| 30 | Potassium channel | Kv4.3 | NA | 17.29 |
| 31 | Serotonin channel | 5-HT_3_ | NA | -15.82 |
| 32 | GABA receptor | α1β2γ2 | NA | 47.01 |
| Transporter class | | | | |
| 33 | Dopamine transporter | DAT | NA | -3.56 |
| 34 | Norepinephrine transporter | NET | NA | 4.45 |
| 35 | Serotonin transporter | SERT | NA | -3.58 |
| Kinase class | | | | |
| 36 | TK | LCK | NA | 0.34 |
| 37 | TK | INSR | NA | 2.46 |
| 38 | TK | VEGFR2 | NA | -3.83 |
| 39 | AGC | ROCK1 | NA | -5.07 |
| Enzyme class | | | | |
| 40 | Cyclooxygenase | COX1 | NA | 14.35 |
| 41 | Cyclooxygenase | COX2 | NA | 30.64 |
| 42 | Cholinesterase | AChE | NA | 13.76 |
| 43 | Phosphodiesterase | PDE3A | NA | 1.23 |
| 44 | Phosphodiesterase | PDE4D2 | NA | 5.75 |
| 45 | Monoamine oxidase | MAO-A | NA | -2.93 |
| Nuclear hormone receptor class | | | | |
| 46 | Androgens | AR | -18.64 | 102.12 |
| 47 | Glucocorticoids | GR | -2.12 | 43.36 |

NA: Not applicable.
